# Microbiological profile of diabetic foot infections in China and worldwide: a 20-year systematic review

**DOI:** 10.3389/fendo.2024.1368046

**Published:** 2024-06-28

**Authors:** Yu-dun Qu, Shuan-ji Ou, Wei Zhang, Jia-xuan Li, Chang-liang Xia, Yang Yang, Jia-bao Liu, Yun-fei Ma, Nan Jiang, Ye-yang Wang, Bo Chen, Bin Yu, Yong Qi, Chang-peng Xu

**Affiliations:** ^1^ Department of Orthopedics, Guangdong Second Provincial General Hospital, Guangzhou, China; ^2^ Division of Orthopedics and Traumatology, Department of Orthopedics, Nanfang Hospital, Southern Medical University, Guangzhou, China; ^3^ Endocrinology Department, Guangdong Second Provincial General Hospital, Guangzhou, China

**Keywords:** diabetic foot, pathogenic bacteria, antibiotics, drug sensitivity test, infection - immunology

## Abstract

**Introduction:**

Pathogens causing diabetic foot infections (DFIs) vary by region globally; however, knowledge of the causative organism is essential for effective empirical treatment. We aimed to determine the incidence and antibiotic susceptibility of DFI pathogens worldwide, focusing on Asia and China.

**Methods:**

Through a comprehensive literature search, we identified published studies on organisms isolated from DFI wounds from January 2000 to December 2020.

**Results:**

Based on our inclusion criteria, we analyzed 245 studies that cumulatively reported 38,744 patients and 41,427 isolated microorganisms. DFI pathogens varied according to time and region. Over time, the incidence of Gram-positive and Gram-negative aerobic bacteria have decreased and increased, respectively. America and Asia have the highest (62.74%) and lowest (44.82%) incidence of Gram-negative bacteria, respectively. Africa has the highest incidence (26.90%) of methicillin-resistant Staphylococcus aureus. Asia has the highest incidence (49.36%) of Gram-negative aerobic bacteria with species infection rates as follows: *Escherichia coli*, 10.77%; *Enterobacter spp.*, 3.95%; and *Pseudomonas aeruginosa*, 11.08%, with higher local rates in China and Southeast Asia. Linezolid, vancomycin, and teicoplanin were the most active agents against Gram-positive aerobes, while imipenem and cefoperazone-sulbactam were the most active agents against Gram-negative aerobes.

**Discussion:**

This systematic review showed that over 20 years, the pathogens causing DFIs varied considerably over time and region. This data may inform local clinical guidelines on empirical antibiotic therapy for DFI in China and globally. Regular large-scale epidemiological studies are necessary to identify trends in DFI pathogenic bacteria.

**Systematic review registration:**

https://www.crd.york.ac.uk/prospero/, identifier CRD42023447645.

## Introduction

Diabetes is a major global health issue that is estimated to affect 592 million people by 2035 (1). Poor diabetes management may lead to diabetic foot infection (DFI), one of the most common and dangerous diabetic complications. It has been estimated that 15% of patients with diabetes will develop DFI in their lifetime (2). DFIs are the leading cause of non-traumatic lower extremity amputations and hospitalizations. Although initially superficial, DFIs can be complicated by osteomyelitis (3), and the costs associated with DFI care are substantial (4). The annual cost of DFI care in England between 2014 and 2015 was estimated at £1 billion (5). According to a previous study, 55% of patients with DFI still had the infection a year after diagnosis, and almost 15% had undergone amputation (6). Therefore, the treatment of DFI is a significant clinical challenge; understanding the microbiological profile is key to tackling this challenge. Prospective and retrospective studies on DFI microbiological profiles have identified a wide variety of pathogens, suggesting the persistent, open, and anatomical characteristics of this illness (7). Methicillin-resistant *Staphylococcus aureus* (MRSA) has been the most common cause of DFI in developed (predominantly North American and European) nations in the last 15 years (8, 9). Additionally, current research has shown that the etiology of DFIs varies significantly by region globally (8, 10, 11). Gram-negative bacteria (GNB) are more prevalent than Gram-positive bacteria (GPB) in warmer Asia and Africa (12). Polymicrobial infections are also major concerns worldwide.

Epidemiological antimicrobial therapies are the current clinical recommendations for DFI management until etiologic agents and their antibiotic sensitivity patterns are identified using wound cultures (9). Therefore, for serious infections, most doctors use broad-spectrum antibiotics to empirically treat the possible pathogens (IWGDF 2019 update) (13). However, DFIs are often caused by antimicrobial-resistant organisms, which may necessitate frequent patient visits to healthcare facilities or antibiotics use (14). Furthermore, owing to the extensive use of broad-spectrum antibiotics and changes in antibiotic resistance genes, the resistance rates of DFI pathogens to antibiotics are increasing substantially (15). Moreover, bacteria frequently form biofilms that resist immune clearance and promote antimicrobial resistance (16). A study detected biofilm in 78.2% of chronic wounds (17). Nearly all current guidelines for DFI management have been prepared in Western countries (9). However, based on recent studies from different regions, antibiotics for DFI treatment may require empirical selection consistent with the local prevalence of pathogenic bacteria. Thus, we conducted a systematic review of published reports from various regions worldwide, particularly China, to determine the microbiological profile of DFIs in different regions and provide a guide for developing or updating local clinical guidelines.

## Research design and methods

### Literature search strategy

Databases, including China Biology Medicine, China National Knowledge Infrastructure, WanFang, VIP, PubMed, MEDLINE, and Web of Science, were searched electronically for articles published on DFI and antibiotic sensitivity testing between January 2000 and December 2020. We searched terms such as diabetic foot, foot ulcer, infection, osteitis, osteomyelitis, diabetic foot osteomyelitis, microbiology, bacteria, fungus, mycoses, anti-infective agents, drug (antibiotic) sensitivity, drug sensitivity test, drug resistance, and antibiotic resistance. Moreover, we searched Google Scholar, Google, and paper references. The titles and abstracts of the initial reports collected were independently reviewed by two authors, who evaluated the relevant papers’ complete texts for eligibility. Articles with missing full text were obtained directly from their authors.

### Inclusion and exclusion criteria

Relevant studies were to report a clinical microbiological examination of bacterial strains and medication sensitivity in diabetic foot ulcers, with a minimum sample size of 10. We excluded duplicate reports, articles that did not satisfy our inclusion criteria, single case reports, posters, subgroup analyses, or theses that had previously been included. As part of our search strategy, we used the Preferred Reporting Items for Systematic Reviews and Meta-Analyses (PRISMA) method (18).

### Data abstraction and quality appraisal

Publication year, first author, research location, study design, research period, number of patients, patient’s age and sex, diabetes duration, number and distribution of pathogenic bacteria, and drug sensitivity were abstracted onto standardized forms. Two authors independently extracted and assessed the quality of the articles.

### Data classification and summary

We grouped research by year(s) of study or data collection and not by the publishing date. We divided the sum of a bacterial species by the total number of bacteria to get the percentage of isolates. All GNB and GPB were tested for antibiotic sensitivity. We compared research of the most recent 5 years (2016–2020) with those of the 20-year period (2001–2020) to examine changes in microbiological profiles and antibiotic sensitivity trends.

### Data and resource availability

The datasets generated during and analyzed in the current study are available from the corresponding author upon reasonable request.

## Results

### General characteristics of included studies

**Figure 1:**
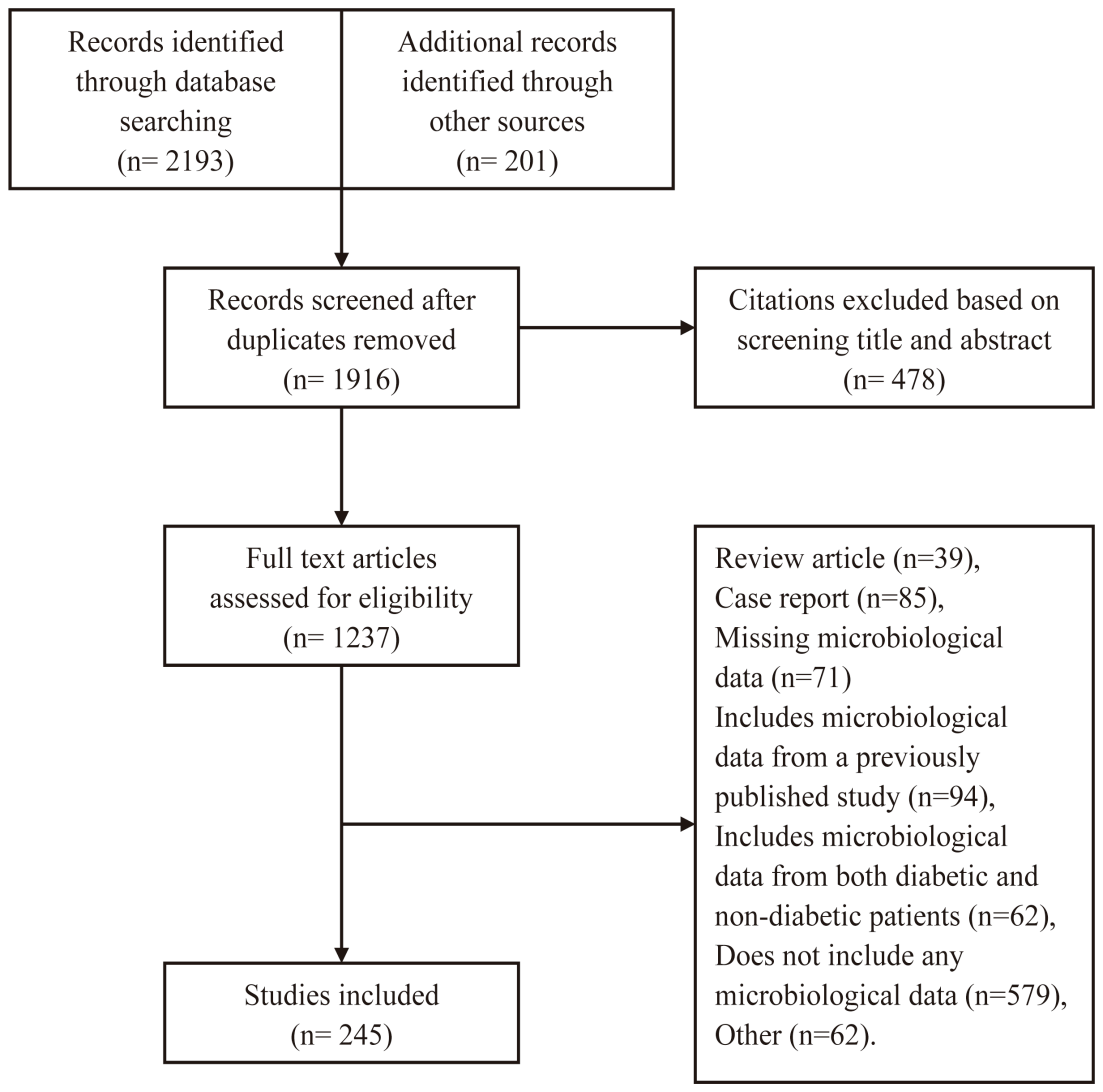
Flow chart of eligibility selection.

We identified 2394 studies (**Figure 1**). Of these, 245 (225 journal articles and 20 theses) from China’s mainland met our inclusion criteria: 195 and 50 were performed in tertiary and secondary care hospitals, respectively. The studies involved 38,744 individuals with 41,427 bacterial isolates from foot wounds.

Methodology differed greatly between the studies: 24 (9.80%) were prospective, whereas the rest were retrospective, and 235 (95.92%) involved inpatients, one (0.41%) included only outpatients, seven (2.86%) included both inpatients and outpatients, and two (0.82%) did not describe the setting. Additionally, wound sampling methods varied: 184 (75.10%) performed swab sampling alone, while 51 (20.82%) of the investigations performed both swab and deep tissue sampling. Of the remaining studies, four (1.63%) performed swab, deep tissue, and bone sampling, four (1.63%) performed deep tissue sampling alone, one (0.41%) performed only bone sampling, and one study did not specify the wound sampling method. While all the studies cultured specimens aerobically, only eight (3.27%) performed anaerobic cultures. Antibiotic sensitivity results were reported by 215 (87.76%) of the studies.

**Supplementary Table S1** presents selected demographic and clinical data obtained from the studies. **Supplementary Figure 1** illustrates the provincial distribution of 41,427 bacterial isolates.

### Trends of various microorganisms during the three study periods

**Figure 2:**
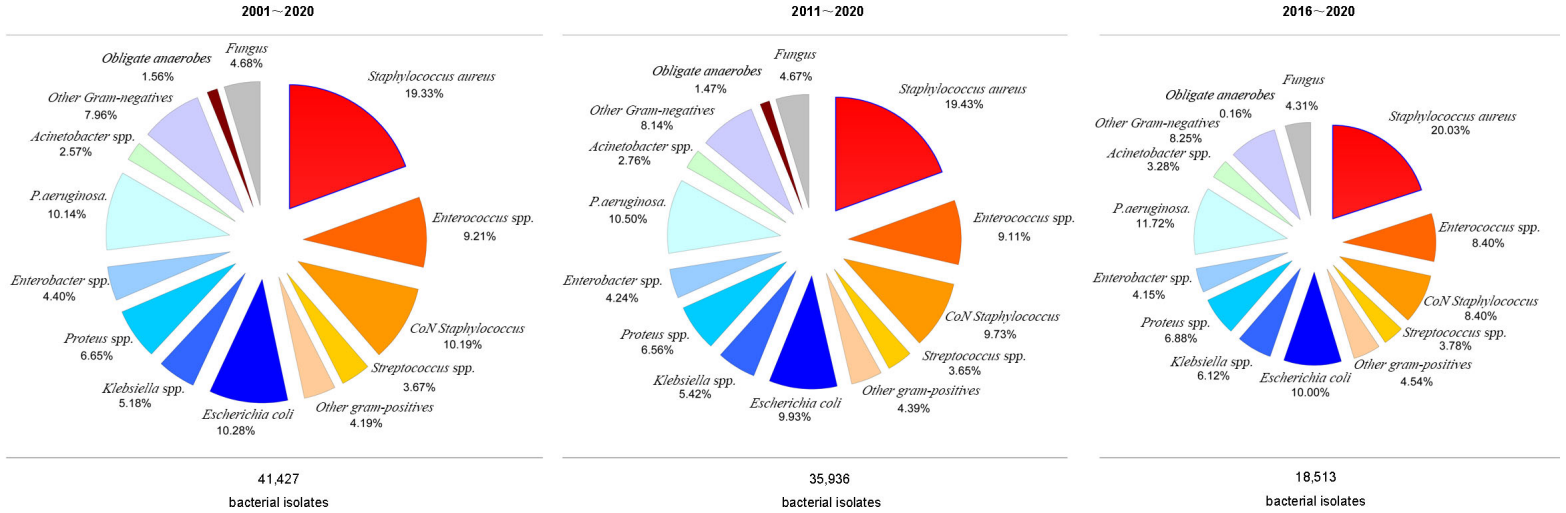
The distribution of microorganisms from 2001 to 2020, 2011 to 2020, and 2016 to 2020.

**Figure 2** presents the prevalence rates of different bacteria over the three study periods. GNB and GPB were equally prevalent between 2001 and 2020 (46.58% vs. 47.18%) and 2011–2020 (46.32% vs. 47.54%). However, GNB and GPB incidences differed considerably between 2016 and 2020 (45.16% vs. 50.38%).

Although *Staphylococcus aureus* isolation rates were similar (20.03, 19.43, and 20.05%) across the 20, 10, and 5-year study periods, MRSA isolation rates increased significantly (8.24, 8.51, and 11.56%, respectively). GPB incidence decreased during the three study periods. However, *Enterococcus* spp. and *Coagulase-negative staphylococcus* isolation rates reduced across all three study periods (**Figure 2**).

**Figure 3:**
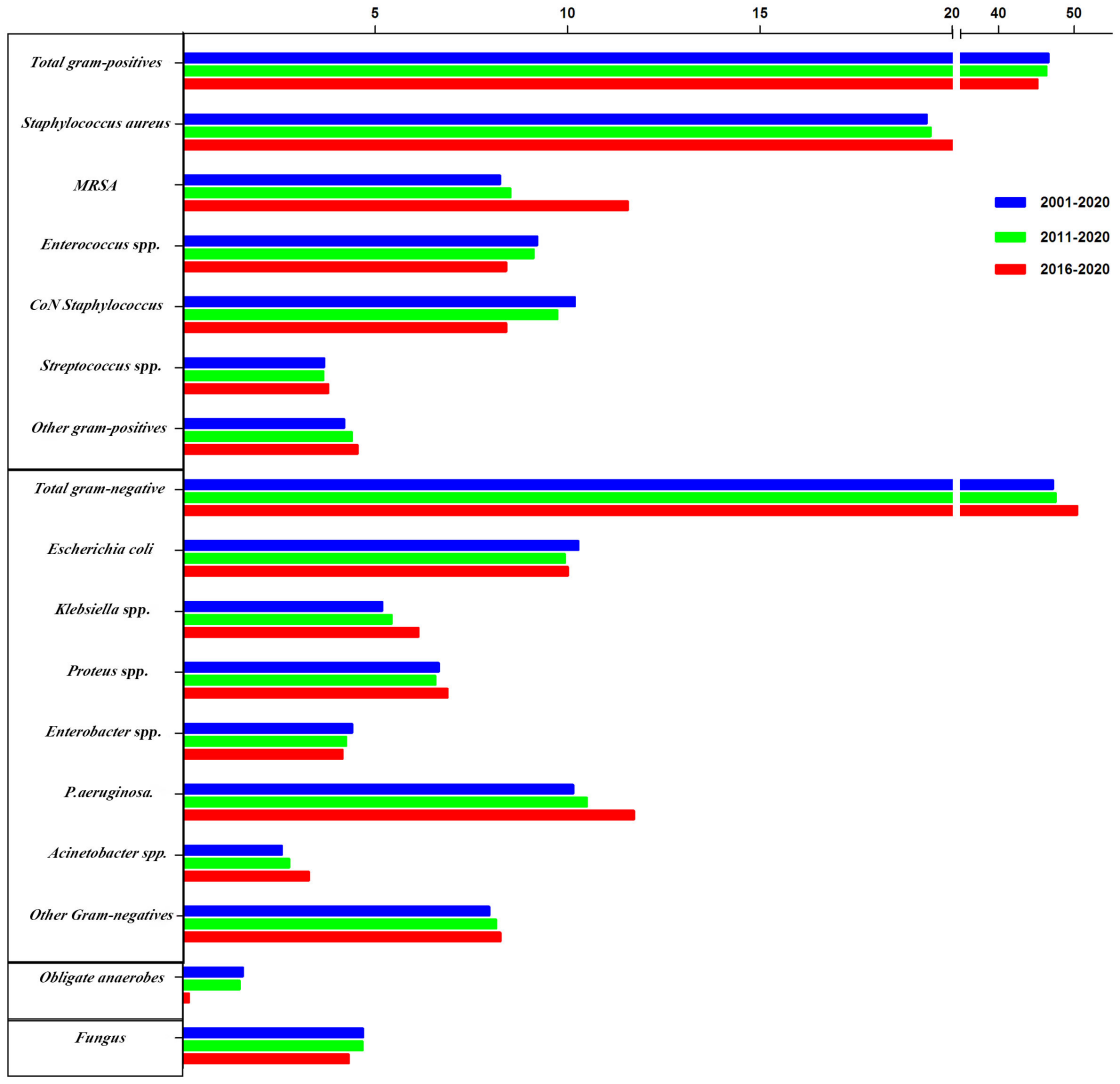
The trends of pooled rates of microorganisms from 2001 to 2020, 2011 to 2020, and 2016 to 2020.

Furthermore, GNB incidence increased during the three study periods, with increased isolation rates of *Klebsiella* spp. (5.18%, 5.42%, and 6.12%, respectively), *Pseudomonas aeruginosa* (10.14%, 10.50%, and 11.72%, respectively), and *Acinetobacter* spp. (2.57%, 2.76%, and 3.27%, respectively), whereas the *E. coli* isolation rate decreased across the three periods (10.28%, 9.93%, and 10.00%, respectively). Similarly, the isolation of obligate anaerobes and fungal species decreased across the three periods, while the incidence of obligate anaerobes decreased significantly between 2016 and 2020 (**Figure 3**).

### Geographical distribution of various microorganisms

Eastern and Western China division was based on the provinces of the patients, with the Hu Line (Heihe-Tengchong Line) set as the boundary. The incidence of GPB and fungal species tended to be higher in the Western region than in Eastern China. Conversely, the incidence of GNB and obligate anaerobes was higher in the Eastern region than in Western China. Moreover, MRSA incidence was higher in Eastern China, while *E. coli*, *Proteus* spp., and *Enterobacter* spp. incidence was higher in Western China (**Supplementary Figure S2**; **Supplementary Table S2**).

Northern and Southern China division was based on the provinces of the patients, with the Tsinling Mountains–Huai River set as the dividing line. The incidence of GPB and fungal species tended to be higher in Southern China than in Northern China. Conversely, the incidence of GNB and obligate anaerobes tended to be higher in Northern than in Southern China. Moreover, MRSA incidence was higher in Northern China, while *E. coli* and *Proteus* spp. incidence was higher in Southern China (**Supplementary Figure 3**; **Supplementary Table S3**).

**Figure 4:**
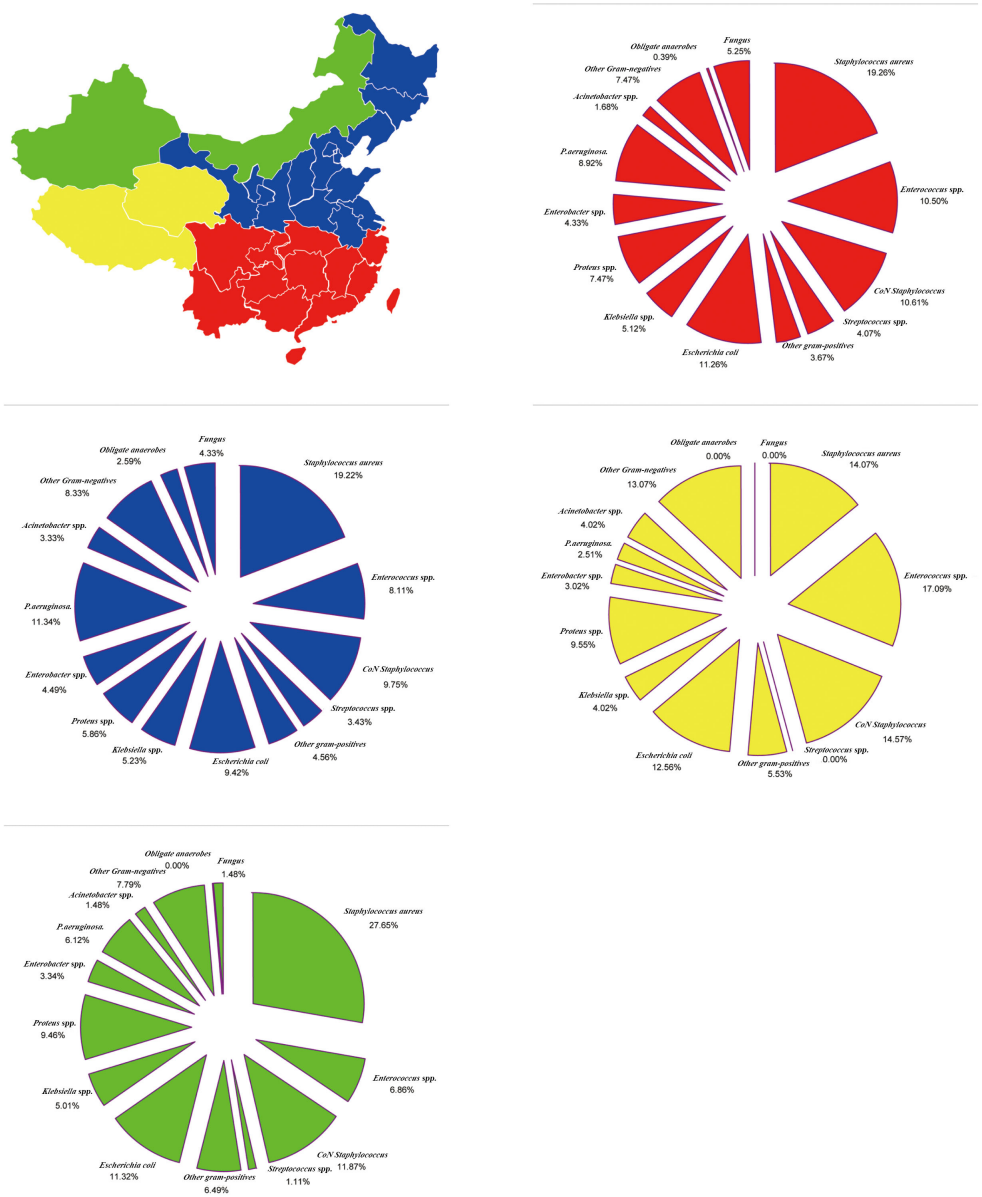
Region-Specific pooled rates of microorganisms from diabetic foot infections in four major geographical regions of China during the entire 20-year period.

The Tsinling Mountains–Huai River line divided China into two, forming the four major geographical regions of China based on the obvious climatic differences between the north and south. The incidence of GPB (53.99%) and MRSA (28.19%) tended to be the highest in Northwest China. On the other hand, the incidence of GNB (48.74%) and *E. coli* (12.56%) tended to be the highest in the Tibetan Plateau area of China. Moreover, the incidence of obligate anaerobes (2.59%) tended to be highest in North China, while the incidence of fungal species (5.25%) was highest in South China (**Figure 4**; **Supplementary Table S4**).

**Figure 5:**
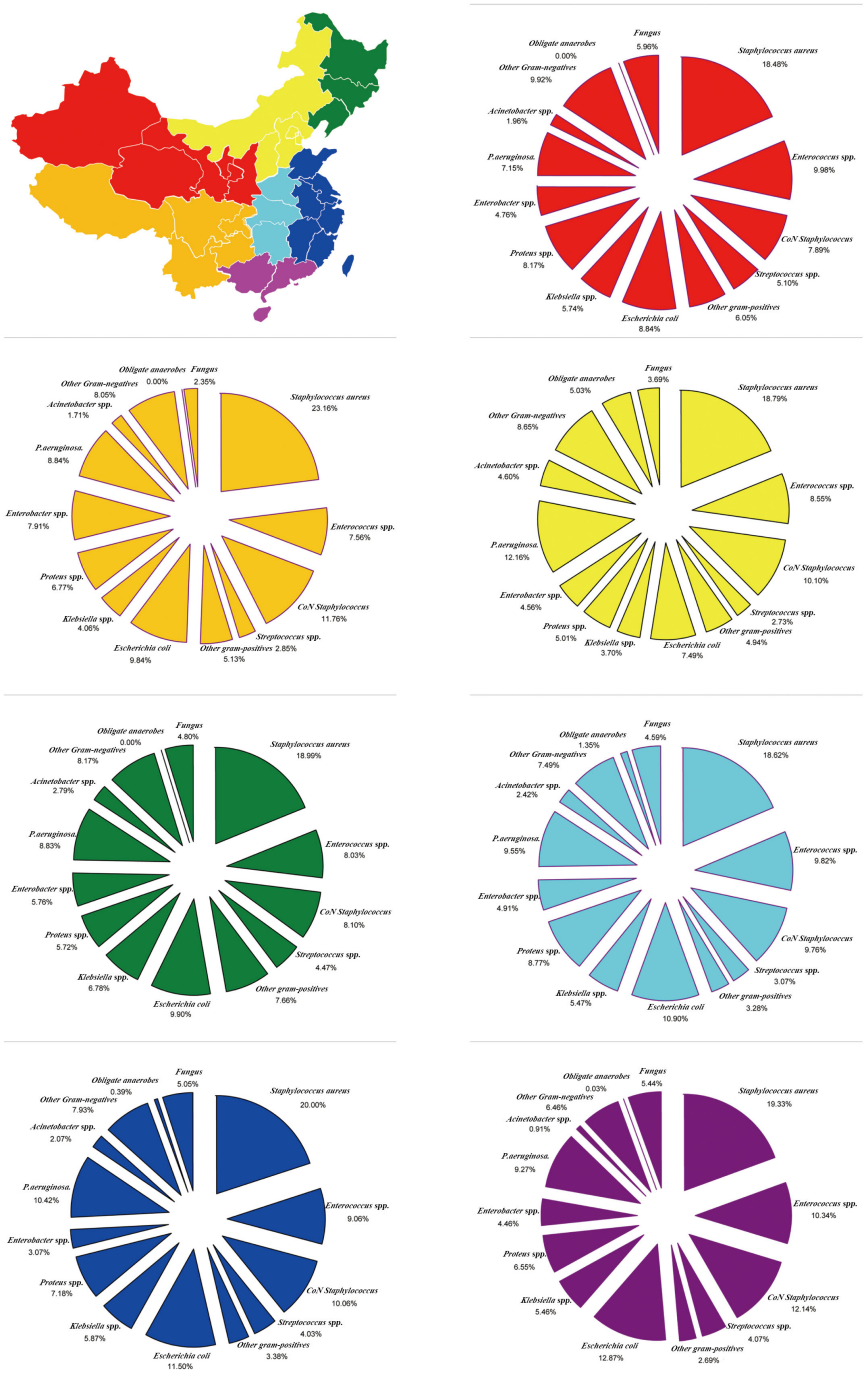
Region-Specific pooled rates of microorganisms from diabetic foot infections in seven major geographical regions of China during the entire 20-year period.

China is divided into seven regions according to its physical geography. GPB incidence was the highest (50.46%) and lowest (44.55%) in Southwest China and Central China, respectively; however, MRSA incidence (19.01%) and GNB (49.51%) were the highest in Central China. The incidence of *E. coli* was the highest in South China (12.87%), *Klebsiella* spp. in the Northeast (6.78%), *Proteus* spp. in Central China (8.77%), *Enterobacter* spp. in the Southwest (7.91%), *P. aeruginosa* in North China (12.16%), and *Acinetobacter* spp. in North China (4.60%). Moreover, obligate anaerobe incidence was the highest in North China (5.03%), and fungal species incidence was the highest in Northwest China (5.96%) (**Figure 5**; **Supplementary Table S5**).

### Distribution of microorganisms in different Asian regions

Based on the theory that antibiotic selection is informed by the type of microbiological profile in Asia, this study examined the composition and susceptibility of bacterial isolate in diabetic foot infection (DFI) samples from Asian centers. Using 315 Asian studies, we evaluated 54,161 tissue and bone samples from 48,241 individuals.

**Figure 6:**
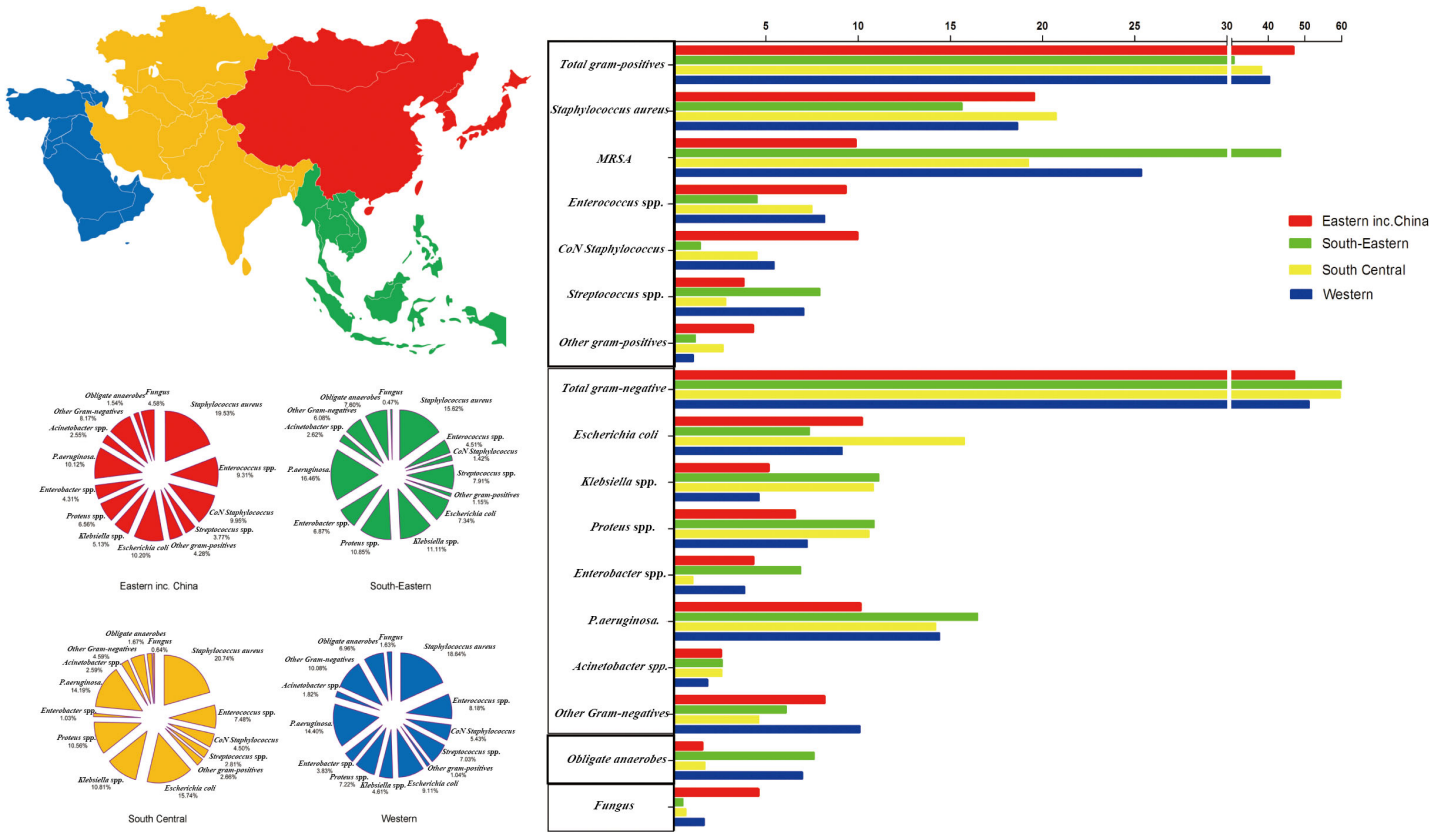
Distribution of microorganisms across four Asian regions and the trends of pooled rates of microorganisms.

Overall, the incidence of GPB and Fungi in other Asian countries compared with that in China (GPB: 44.82% and 46.58%, respectively; Fungi: 3.78% and 4.68%, respectively) was lower. The incidence of GNB and obligate anaerobes in other Asian countries compared with that in China (GNB: 49.36 and 47.18%, respectively; 2.04% and 1.56%, respectively) tended to be higher (**Figure 6**). Notably, MRSA isolation rates were significantly lower in China than in other Asian countries (8.24 and 12.82%, respectively).

Asia is divided based on physical geography into Eastern, Southeast, South Central, and Western Asia regions. The incidence of GPB in Eastern Asia (including China) was the highest (46.84%), whereas GPB incidence in Southeast Asia was the lowest (30.61%); however, MRSA incidence in Southeast Asia tended to be the highest (43.29%). GNB incidence (61.32%) in Southeast Asia tended to be the highest. The incidence of *E. coli* (15.74%) in South Central Asia and *Klebsiella* spp. (11.11%), *Proteus* spp. (10.85%), *Enterobacter* spp. (6.87%), *Pseudomonas aeruginosa* (16.46%), and *Acinetobacter* spp. (2.62%) in Southeast Asia tended to be the highest. Moreover, obligate anaerobe incidence in Southeast Asia trended to be the highest (7.60%), and fungal species incidence in Eastern Asia (including China) tended to be the highest (4.58%) (**Figure 6**; **Supplementary Table S6**).

### Distribution of microorganisms in different world regions

Based on DFI samples from different centers worldwide, we analyzed the global bacterial isolate profile from 2000–2020. Asian microbiological profile differs from that of other continents and should inform empirical antimicrobial selection. Between 2000 and 2020, 359 studies reported 56,592 cases (38,744 from China) and 67,151 bacterial isolates (41,427 from China).

**Figure 7:**
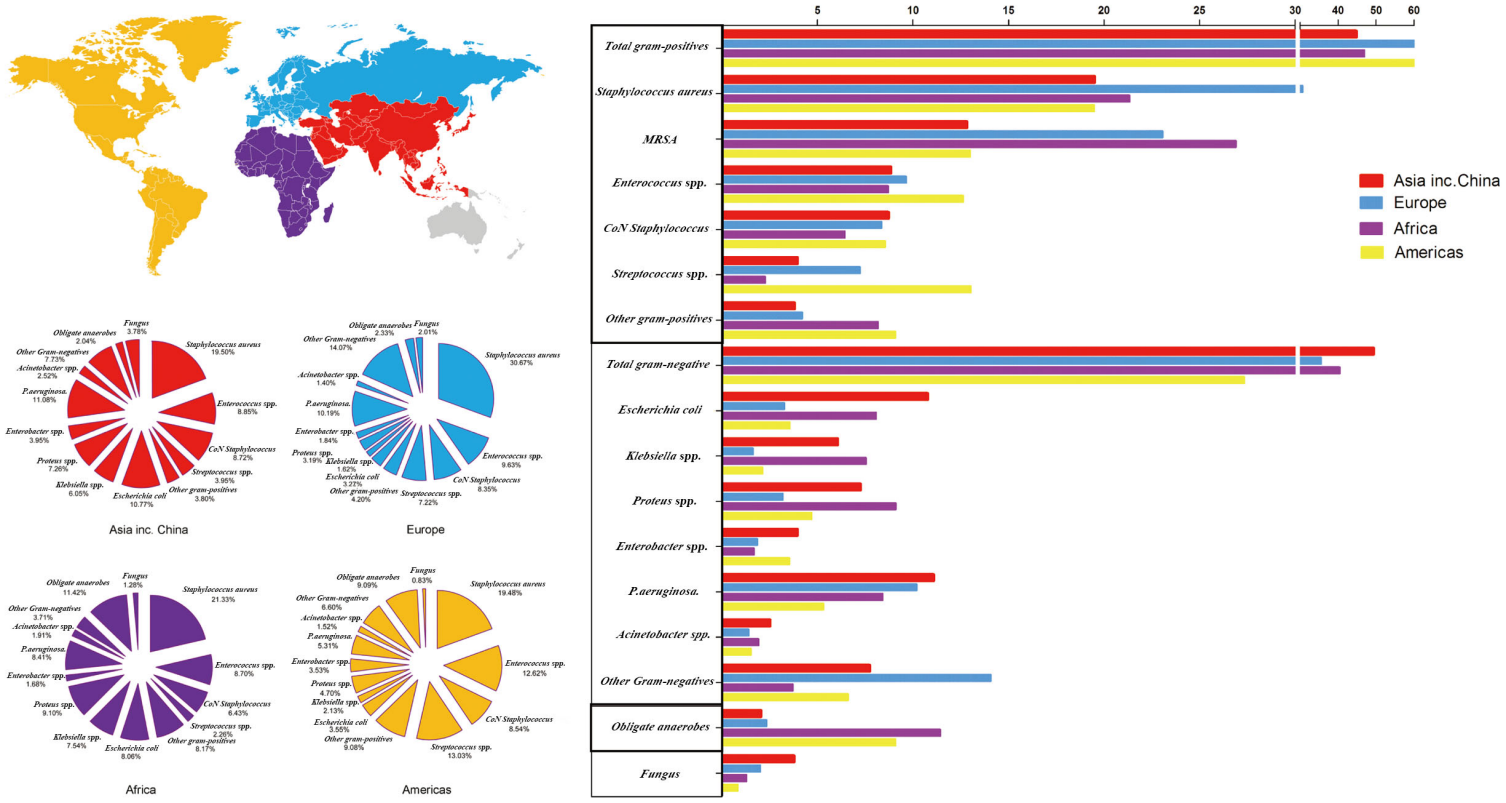
Distribution of microorganisms across four world regions and the trends of pooled rates of microorganisms.

The isolation rates of GPB in DFI were greatest in America (62.74%) and lowest in Asia, including China (44.82%). Africa had the most isolated MRSA (26.90%). GNB (49.36%) were mostly common in Asia. In Asia, *E. coli* (10.77%), *Enterobacter* spp. (3.95%), *P. aeruginosa* (11.08%), *Acinetobacter* spp. (2.52%), and *Klebsiella* spp. (7.54%) were the most common. In Africa, *Klebsiella* spp. (11.11%) and *Proteus* spp. (9.10%) were the most common. The incidence of obligate anaerobe and fungi was highest in Africa (11.42%) and Asia (3.78%), respectively (**Figure 7**; **Supplementary Table S7**).

### Antibiotic susceptibility of isolates from DFI in China

**Supplementary Table S8** presents antibiotic susceptibility for all GPB isolates. Vancomycin, linezolid, and teicoplanin were effective against GPB. Enterococcus spp. was most sensitive to vancomycin (92.13%), linezolid (88.02%), and teicoplanin (86.97%). CoN staphylococci were particularly sensitive to vancomycin (96.08%), linezolid (92.77%), and teicoplanin (90.37%). Vancomycin (96.36%), linezolid (79.90%), levofloxacin (75.95%), teicoplanin (70.97%), penicillin (72.02%), ampicillin-sulbactam (72%), and rifampicin (70%) were the most effective antimicrobials against *Streptococcus* spp.

**Supplementary Table S9** presents GNB antimicrobial sensitivity parameters. No antimicrobial agent inhibited all GNB strains. Imipenem was the most effective agent against *E. coli, Klebsiella* spp.*, Proteus* spp.*, Enterobacter* spp.*, P. aeruginosa, and Acinetobacter* spp., with 94.70%, 93.66%, 88.24%, 90.61%, 81.97%, and 68.42% of strains susceptible, respectively.

The isolation rate of anaerobes was 1.56% (645 strains from 41,427 samples). However, no study reported culture results for strains of obligate anaerobes.

### Worldwide antibiotic susceptibility patterns of isolates obtained from DFIs

Based on the assumption that the global microbiological profile affects antimicrobial selection, this research examined antibiotic susceptibility trends in DFI samples from different centers worldwide. **Supplementary Table S10** presents antibiotic susceptibility data of all GPB isolates. Vancomycin was the most effective against GPB in Asia (98.05%), Africa (100%), Europe (100%), and America (74.07%). Vancomycin was the most effective against *Enterococcus* spp. in Asia (91.96%), Africa (92.81%), and Europe (100%). Vancomycin was the most effective against CoNS, with 96.19% and 100% sensitive strains in Asia and Africa, respectively. Vancomycin was the most effective against *Streptococcus* spp., with 94.83%, 100%, and 16.67% susceptible strains in Asia, Europe, and America, respectively.

**Supplementary Table S11** presents GNB antimicrobial sensitivity values. No antibiotic was 100% effective against GNBs; however, imipenem was the most effective against *E. coli*, with 92.57%, 100%, and 40% susceptible strains in Asia, Africa, Europe, and America, respectively. Imipenem was the most effective against *Klebsiella* spp., with 93.27%, 98.77%, 100%, and 88.77% susceptible strains in Asia, Africa, Europe, and America, respectively. Imipenem was the most effective against *Proteus* spp., with 90.40%, 97.22%, 71.43%, and 78.79% susceptible strains in Asia, Africa, Europe, and America, respectively. Imipenem was the most effective against *Enterobacter* spp., with 91.43%, 96.00%, 98.22%, and 66.67% of DFI-susceptible strains in Asia, Africa, Europe, and America, respectively. Impenem was the most effective against *P. aeruginosa*, with 83.59%, 97.06%, 75.18%, and 79.31% strains susceptible strains in Asia, Africa, Europe, and America, respectively. Imipenem was effective against *Acinetobacter* spp., with 69.72%, 37.84%, and 92.315 strains in Asia, Africa, and America, respectively.

## Discussion

There are four major findings from our analysis. First, between 2001 and 2020, the incidence of DFI caused by GPB in China decreased, whereas the incidence of DFI due to GNB increased. Moreover, the prevalence rates of DFI pathogenic bacteria varied significantly across different regions of China. Second, during this period, the infection rates of MRSA and *Pseudomonas aeruginosa* increased significantly, whereas the infection rates of obligate anaerobic bacteria and fungi decreased. Third, antibiotic resistance and multiple-resistant organisms are increasingly becoming serious problems, and imipenem and vancomycin are currently the main drugs active against GNB and GPB, respectively. Finally, contrary to that in Asia, DFI in developed countries in Europe and America are predominantly caused by GPB.

When comparing the total period: 2001–2020, 2011–2020, and the most recent years, 2016–2020, we found that GNB has higher isolation rates than GPB from DFIs (47.18 vs. 46.58%, 47.54 vs. 46.32%). According to studies conducted in Western countries, GPB are the predominant organisms isolated from DFI (8, 19). However, over the last 20 years, GNB have dominated diabetic foot-infected wounds in China (20). Several studies have reported similar observations; for example, reports from India showed an increased prevalence of GNB in DFIs (21). Owing to the changing microbiological profile of DFI pathogenic bacteria, Chinese tertiary care hospitals must reevaluate empirical treatment for patients with DFI. Based on infection severity and microbiological data, such as culture or Gram-stained smear results, treatment may be adjusted.

Mostly, when infections are polymicrobial, GPB, mainly *S. aureus*, play a dominant causative role. Antibiotic-resistant organisms, especially MRSA, were isolated from 10–32% of patients with DFIs, eventually leading to a higher treatment failure rate (22). However, in our analysis of the *S. aureus* strains, MRSA prevalence was 8.24% in China over the past 20 years. The prevalence rate of MRSA in China was similar to that in other Asian countries, such as India, Kuwait, Malaysia, and Korea, where it was 7.1–14.3% (11). Notably, MRSA prevalence increased in the most recent 5 years compared to the previous periods (8.24% in 2001–2020, 8.51% in 2011–2020, and 11.56% in 2016–2020). This recent rise in MRSA prevalence is consistent with other reports (23). The increasing bacteria resistance and the adverse effects of currently available anti-MRSA agents have limited treatment options for patients with DFI. The problem associated with MRSA continues to increase despite the precautions taken to prevent MRSA spread. Therefore, a multicenter study on MRSA prevalence in diabetic foot ulcers and how it can be reduced in diabetic foot clinics is necessary.

Another finding of note in our study is the increasing prevalence of *Pseudomonas aeruginosa* infection in Chinese patients with DFI (10.14% in 2001–2020, 10.50% in 2011–2020, and 11.72% in 2016–2020). *Pseudomonas* spp. is the most frequently isolated bacteria among the GNB (47.18%), followed by *Enterobacter* spp. (10.28%). Published data showed that the isolation rate of *Pseudomonas aeruginosa* from DFIs was 5.8% (24) and 7.8% (25) in North America, approximately 13–20% in India (26), and approximately 13.7–14.9% in Turkey (23). Therapy against *Pseudomonas* spp. is usually different from that against other organisms, resulting in increasing pseudomonas infection in diabetic foot clinics. Therefore, clinicians need to know the local prevalence of these species.

The distribution of pathogens in DFI ulcers has evolved. Previous studies have reported that compared to GNB, GPB is predominantly isolated in DFI (27), and a survey conducted in Southern China between 2009 and 2014 showed GPB and GNB infection rates of 54% and 48.8%, respectively (28). However, in our study, we found that the endemic flora of DFI changed, and the percentage prevalence of GNB (47.18%) was slightly higher than that of GPB (46.58%), whereas no significant difference was observed over the past 20 years. GNB prevalence in Eastern and Northern China was higher than that in Southern and Western China. In a recent study, GNB prevalence in DFI increased with temperature and humidity (29). A study of DFI in Chinese patients showed similar results to our results, reporting that the prevalence of GPB was 43.4% compared with 52.4% for GNB (20).

Our systematic study revealed that the prevalence of MRSA, *P. aeruginosa*, obligate anaerobes, and fungal species differed between Northern–Southern and Eastern–Western regions. Moreover, the spectrum of microbe isolated from foot ulcers among patients with diabetes differed in four or seven major geographical regions of China. Notably, the prevalence of fungal species in Southern China was higher than that in other regions. These results could be explained by differences in climatic factors, such as temperature and humidity based on geography, which influence the spectrum of bacteria infecting foot ulcers (29). Additionally, diet and constitutional factors may influence the incident bacterial spectrum. The composition of bacteria present in DFI is related to ulcer duration and previous antibiotic exposure (30). Poor hygiene, delayed diagnosis and treatment, and inappropriate use of empirical antibiotics in DFI may contribute to changes in the bacterial spectrum (31). Therefore, the different distribution of bacteria in DFI could be associated with socioeconomic status, hygiene, and the use of footwear.

Evidence shows that bacteria in DFI vary across Asia. GNB infections are more frequent in Southeast Asia than in East Asia. Moreover, Southeast Asia is a breeding ground for many other bacteria, such as MRSA, *Klebsiella* spp.*, Proteus* spp.*, Enterobacter* spp.*, P. aeruginosa, and Acinetobacter* spp. Most studies conducted in Southeast Asia reported a higher prevalence of GNB in DFIs than in other regions, and at least half of them reported that more than two-thirds of the isolates were GNB (31). GNB (29.7%) outnumbered GPB (19.4%) in a retrospective cross-sectional investigation of 434 patients with DFI from January 2010 to December 2019 (32). These findings are supported by previous studies in Malaysia and Bermuda (33). In contrast, while studies in Western countries have identified most of the bacteria isolated from DFIs as GPB (10), GNB are prevalent in DFIs in developing countries in the Southeast Asia region, and nine of 10 studies in the Southeast Asian region reported a higher prevalence of GNB in DFIs (34).

Environmental factors may explain the higher rate of GNB infections in developing countries in Southeast Asia. Southeast Asia mainly has a tropical rainforest climate, which is hot and rainy all year round, and GNB are mostly heat-resistant organisms more likely to propagate in a hot and humid environment; hence, the closer the location to the equator, the higher the probability of GNB being prevalent. Hygiene practices, such as perianal washing with water after defecation, often lead to contamination of the hands with fecal flora rich in GNB, which can lead to infection (10). Additionally, our study found an increasing trend of DFIs caused by obligate anaerobes and fungi in Southeast and East Asia, respectively, although no other literature has confirmed this.

The DFI pathogenic bacterial spectrum varies greatly across different regions worldwide. Recent studies suggest that GNB prevalence in DFI is as high as 70% in developing countries (21, 35). Studies from several Asian nations have reported varied frequencies of isolated *Staphylococcus aureus* from DFIs of 11.8–26% (8, 23, 35, 36). GNB, GPB, and obligate anaerobes were responsible for 51.2%, 32.3%, and 15.2% of DFIs, respectively, in a study of 440 patients in Kuwait (8). In a recent research conducted in India, anaerobic bacteria, GPB, and GNB accounted for 15.1%, 33.3%, and 51.4% of foot infections (21). Studies conducted in affluent nations (mostly Europe) revealed that *Staphylococcus aureus* accounts for 30.1–48.8% of isolates as the etiological agent in DFIs (12, 24). A major U.S. multicenter investigation found that GPB and GNB accounted for 77% and 21.2% of DFIs, respectively (24). Similarly, in Portugal, 66%, 19%, and 13.6% of DFIs were caused by GPB, GNB, and obligate anaerobes, respectively (12). MRSA prevalence rates in Asia and Europe differed. India, Kuwait, Malaysia, and South Korea have 7.1–14.3% MRSA prevalence (36). In the last 5 years, the incidence of MRSA-induced DFI has declined (7.8% between 1989 and 2011 and 5.7% between 2007 and 2011) (23); however, an opposite trend was observed in Europe. In 2003, 30.2% of DFI in Manchester, United Kingdom, had MRSA isolates, almost doubling the recorded incidence 3 years earlier (37). Studies in France, Spain, and America reported 12–20% MRSA-induced DFI (37). A meta-analysis found a high prevalence of GPB isolates in high-income countries and a high prevalence of GNB isolates in low- and middle-income countries (19). Cultural, geographical, and climatic variables may explain the large microbiological disparity between DFIs in Western (North American and European) and Asian nations (including China). Additionally, specimen collection, transport, and analysis, as well as antibiotic dosage and type may change DFI-causing flora.

We compared the drug sensitivity of DFI isolates from China, Europe, America, and Africa. Amikacin was active against 70% of GNB and imipenem against 90% (excluding Acinetobacter spp.). Notably, diabetic foot due to Pseudomonas is a particular and worsening problem. In our analysis, the rates of sensitivity of *Pseudomonas aeruginosa* strains to carbapenems, amikacin, and piperacillin-tazobactam were 81.97%, 71.25, and 68.54%, respectively, which is consistent with the findings of other studies (38). Imipenem and piperacillin-tazobactam were used to treat DFI caused by GNB, with imipenem being the most effective (>80%) (8, 38). Cefoperazone-sulbactam and amikacin are commonly used in Asia, including China (39). In contrast, most antibacterial medications are not active against GNB, particularly in China, suggesting that antibiotic misuse in treating GNB infections is a problem in China; therefore, drug regulation is needed.

When broad-spectrum antibiotics are used excessively, more harmful bacteria develop resistance to several medicines and cause hospital-acquired illnesses. The rise of multidrug-resistant pathogens necessitates careful empiric antibiotic treatment. The emergence of superbugs, which are resistant to all available antibiotics, is a serious threat (40).

Compared with previous studies, our study has some advantages. First, the duration is long. Our study included the most relevant clinical studies between 2000 and 2020. Simultaneously, the changing trend of DFI pathogenic bacteria in China was analyzed over three periods. Second, the included studies were widely distributed. Our study analyzed changes in the DFI microbiota worldwide, providing insights into the differences in the emergence of dominant pathogens across different geographic regions. Third, our analysis was comprehensive. In China alone, we included 245 relevant papers and extracted detailed data on 17 DFI pathogens. However, our study had some limitations, the most important being that our results were based on published data and not prospective trials. The published data represent only a sample of all patients with DFI. Second, this study covers most regions of the world; however, owing to differences in technology, equipment, and clinical microbiology laboratory practice in different regions, the accuracy of bacterial detection and drug sensitivity tests may vary. Third, we counted the proportion of pathogenic bacteria in different periods and different regions but did not do further statistical analysis, we focused on the change in the proportion trend. Fourth, this study is a literature review; all studies were retrospective. Data integrity and homogeneity cannot be guaranteed, which may affect the reliability of results.

In conclusion, our findings have important clinical implications, particularly regarding the selection of empiric treatment for DFIs. Based on the epidemiological trend of DFI pathogenic bacteria in different areas, different antimicrobial drugs should be considered. Antimicrobial resistance and multidrug-resistant bacterial infections pose great challenges. The high incidence of MRSA and *Pseudomonas aeruginosa* suggests that traditional approaches to empirical treatment may need to be reevaluated. Empirical antibiotic treatment should naturally include MRSA and Pseudomonas spp. therapy for patients with severe DFI, pending culture and sensitivity findings. It also requires more detailed public health policies to prevent the emergence of more resistant bacteria as a result of drug abuse, making treatment more difficult. Therefore, we suggest that treatment guidelines for DFI should be regularly updated and dynamically adjusted, rather than being kept constant. The establishment of regional and global database management centers, global surveillance, and real-time early warning, such as MRSA, to help formulate better public health policies.

## Data availability statement

The original contributions presented in the study are included in the article/**Supplementary Material**, further inquiries can be directed to the corresponding authors.

## Author contributions

Y-DQ: Conceptualization, Data curation, Formal analysis, Investigation, Methodology, Project administration, Resources, Software, Validation, Visualization, Writing – original draft, Writing – review & editing. S-JO: Conceptualization, Data curation, Investigation, Methodology, Project administration, Software, Validation, Writing – original draft. WZ: Data curation, Formal analysis, Funding acquisition, Methodology, Project administration, Resources, Software, Visualization, Writing – original draft. J-XL: Investigation, Software, Writing – original draft. C-LX: Data curation, Investigation, Writing – original draft. YY: Conceptualization, Formal analysis, Writing – original draft. J-BL: Resources, Validation, Visualization, Writing – original draft. Y-FM: Conceptualization, Formal analysis, Investigation, Methodology, Project administration, Writing – original draft. NJ: Conceptualization, Data curation, Formal analysis, Project administration, Software, Writing – original draft. Y-YW: Conceptualization, Data curation, Formal analysis, Investigation, Methodology, Project administration, Visualization, Writing – original draft. BC: Data curation, Formal analysis, Funding acquisition, Project administration, Resources, Validation, Writing – original draft. BY: Conceptualization, Data curation, Formal analysis, Funding acquisition, Methodology, Supervision, Validation, Writing – original draft. YQ: Conceptualization, Data curation, Funding acquisition, Methodology, Supervision, Validation, Visualization, Writing – review & editing, Writing – original draft. C-PX: Conceptualization, Data curation, Funding acquisition, Methodology, Supervision, Validation, Visualization, Writing – original draft, Writing – review & editing.
