# Supplementary material for: Microbiological profile of diabetic foot infections in China and worldwide: a 20-year systematic review
Source: Front Endocrinol (Lausanne). 2024 Jun 28;15:1368046. doi: 10.3389/fendo.2024.1368046 (PMC11247326; doi:10.3389/fendo.2024.1368046)
Supplement: Supplementary file 1 [file Image_1.pdf]

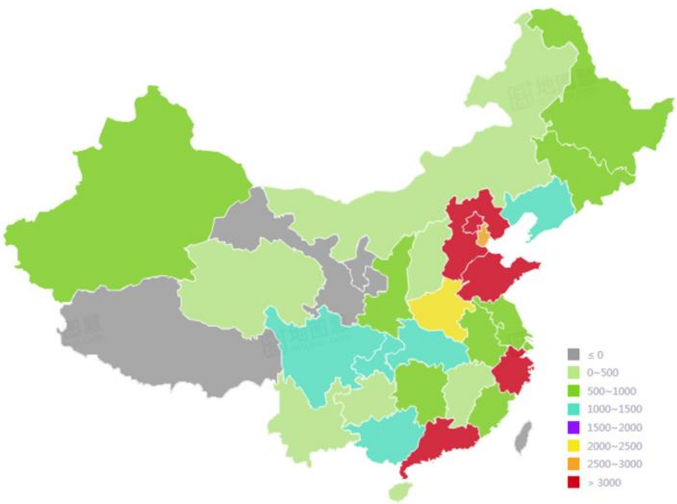

Figure S1. The provincial distribution of the 41,427 bacterial isolates from studies included in the review.

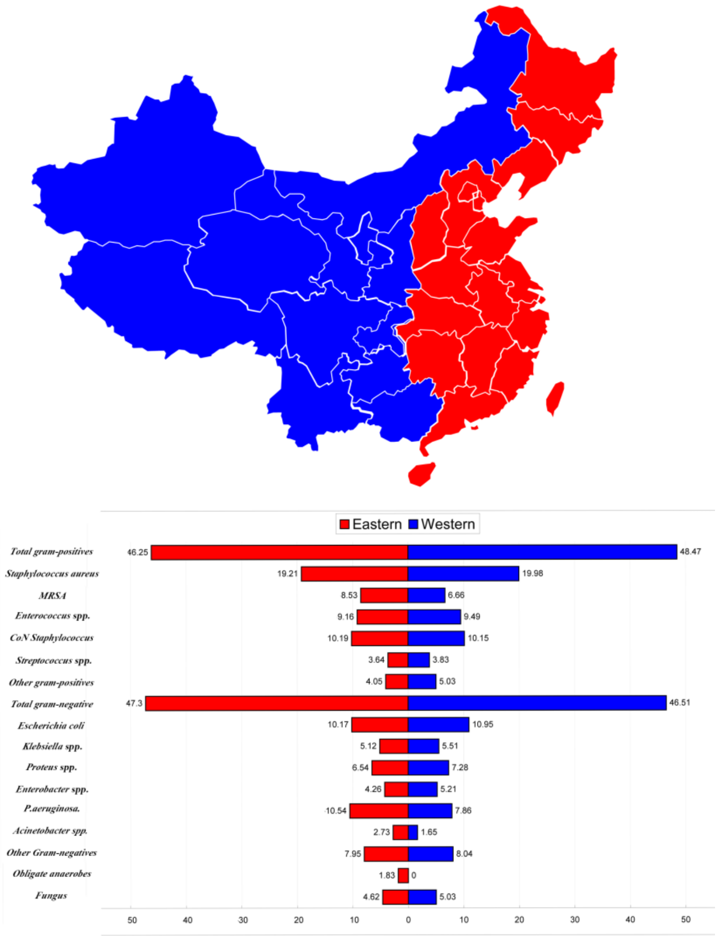

Figure S2. Region-Specific pooled rates of microorganisms from diabetic foot infections between Eastern and Western during the entire 20-year period.

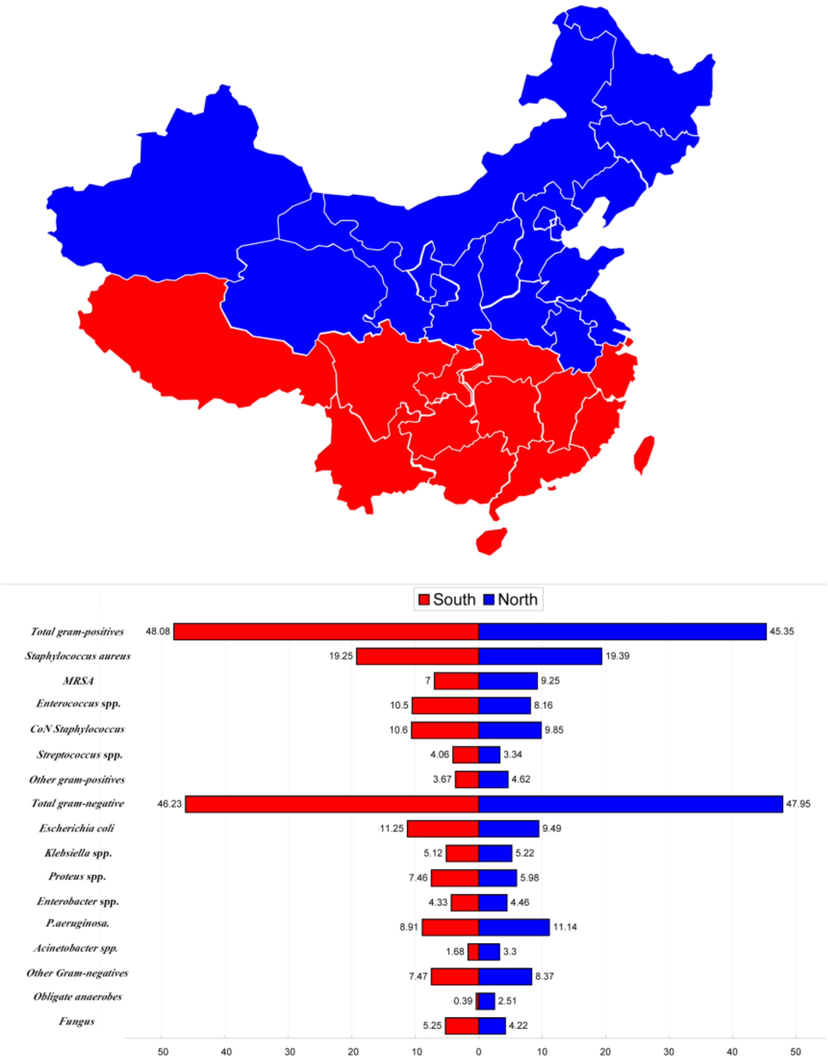

Figure S3. Region-Specific pooled rates of microorganisms from diabetic foot infections between north and south during the entire 20-year period.
