# Supplementary material for: Microbiological profile of diabetic foot infections in China and worldwide: a 20-year systematic review
Source: Front Endocrinol (Lausanne). 2024 Jun 28;15:1368046. doi: 10.3389/fendo.2024.1368046 (PMC11247326; doi:10.3389/fendo.2024.1368046)
Supplement: Supplementary file 2 [file Table_1.docx]

Table S1. Main characteristics of 425 studies included in this systematic review.

| Author,  Year | Dates  of sample  collection | City | Study  design | No  of PT | Sampling  method | Mean age  (years) | Setting | Specimen  culture | Antibiotic sensitivity report |
| --- | --- | --- | --- | --- | --- | --- | --- | --- | --- |
| Chang BC, 2001 | 1997/05~2000/12 | Tianjin | Retrospective | 80 | Swab | 69.2 | Inpatient | Aerobical | Yes |
| Liu L, 2001 | 1990/01~1999/12 | Luoyang | Retrospective | 23 | Swab | 56.2 | Inpatient | Aerobical | Yes |
| Zhang GY, 2001 | 1997/05~1999/10 | Tianjin | Retrospective | 148 | Swab | 61.4 | Inpatient | Aerobical  & Anaerobic | Yes |
| Chao WS, 2002 | 1999/01~2001/12 | Xiping | Retrospective | 18 | Swab | 65.4 | Inpatient | Aerobical | Yes |
| Guo Y, 2003 | 1995/01~2002/12 | Jinan | Retrospective | 93 | Swab | 65.9 | Inpatient | Aerobical | Yes |
| Wang M, 2003 | 1997/05~2001/06 | Shijiazhuang | Retrospective | 40 | Swab | N/A | Inpatient | Aerobical | Yes |
| Li YL, 2004 | 1997/01~2002/12 | Liuzhou | Retrospective | 30 | Swab | N/A | Inpatient | Aerobical | Yes |
| Wang XM, 2004 | 2002/01~2003/12 | Dalian | Retrospective | 46 | Swab | N/A | Inpatient | Aerobical | Yes |
| Zou ZD, 2004 | 1998/03~2002/08 | Dandong | Retrospective | 33 | Swab | 64.3 | Inpatient | Aerobical | No |
| Deng JD, 2005 | 1999/01~2003/12 | Guangzhou | Retrospective | 405 | Swab, Tissue | N/A | Inpatient | Aerobical | Yes |
| Liang YA, 2006 | 2000/07~2004/06 | Beijing | Retrospective | 108 | Swab | 53.7 | Inpatient | Aerobical | Yes |
| Chen LQ, 2006 | 2003/01~2005/12 | Huizhou | Retrospective | 235 | Swab, Tissue | N/A | Inpatient | Aerobical | Yes |
| Lei GD, 2006 | 1997/01~2005/11 | Huaiji | Retrospective | 30 | Swab | N/A | Inpatient | Aerobical | Yes |
| Yang YH, 2006 | 2003/01~2005/06 | Shantou | Retrospective | 72 | Swab | 64.5 | Inpatient | Aerobical | Yes |
| Cai J, 2006 | 2003/08~2005/11 | Shanghai | Retrospective | 45 | Swab | 63.6 | Inpatient | Aerobical | Yes |
| Huang HY, 2007 | 2000/01~2007/06 | Nangning | Retrospective | 56 | Swab | 69.9 | Inpatient | Aerobical | Yes |
| Jiang TJ, 2007 | 2002/06~2006/12 | Changsha | Retrospective | 70 | Swab | 62.3 | Inpatient | Aerobical  & Anaerobic | Yes |
| Xie ZY, 2007 | 2002/01~2006/12 | Putian | Retrospective | 135 | Swab | N/A | Inpatient | Aerobical | Yes |
| Zhu X, 2007 | 2005/06~2007/01 | Changsha | Retrospective | 113 | Swab | 63 | Inpatient | Aerobical | Yes |
| Du W, 2007 | 2003/10~2006/10 | Shanghai | Prospective | 128 | Swab | 75.3 | Inpatient | Aerobical | Yes |
| Jin HW, 2008 | 2000/01~2006/05 | Shaoxing | Retrospective | 95 | Swab, Tissue | N/A | Inpatient | Aerobical | Yes |
| Xu LZ, 2008 | 2003/07~2007/02 | Taizhou | Retrospective | 52 | Swab | N/A | Inpatient | Aerobical | Yes |
| Feng SH, 2008 | 2007/01~2008/01 | Wulumuqi | Retrospective | 90 | Swab | N/A | Inpatient | Aerobical | No |
| He H, 2008 | 2003/01~2005/12 | Qingdao | Retrospective | 129 | Swab | N/A | Inpatient | Aerobical | Yes |
| Han LC, 2008 | 2005/01~2008/06 | Shenzhen | Retrospective | 64 | Swab | 68.8 | Inpatient | Aerobical | Yes |
| Wang SC, 2008 | 2004/01~2006/12 | Linyi | Retrospective | 56 | Swab | 67.1 | Inpatient | Aerobical | Yes |
| Cheng LX, 2008 | 2005/06~2007/06 | Weifang | Retrospective | 32 | Swab | 65.7 | Inpatient | Aerobical | Yes |
| Chen A, 2008 | 2004/08~2007/08 | Zhanjiang | Retrospective | 120 | Swab | N/A | Inpatient  & Outpatient | Aerobical | Yes |
| Lou DJ, 2009 | 2000/06~2007/12 | Shaoxing | Retrospective | 102 | Swab | 65.2 | Inpatient | Aerobical | Yes |
| Feng SH, 2009 | 2005/01~2007/12 | Tianjin | Retrospective | 452 | Swab | 66 | Inpatient | Aerobical | Yes |
| Zhou LJ, 2009 | 2003/01~2007/12 | Dandong | Retrospective | 89 | Swab | N/A | Inpatient | Aerobical | Yes |
| Yang J, 2009 | 2005/01~2008/07 | Hengyang | Retrospective | 68 | Swab | 61 | Inpatient | Aerobical | Yes |
| Niu YG, 2009 | 2005/08~2007/10 | Xintai | Retrospective | 45 | Swab | 56~78 | Inpatient | Aerobical | Yes |
| Shen M, 2009 | 2000/01~2007/01 | Anqing | Retrospective | 84 | Swab | 32~81 | Inpatient | Aerobical | Yes |
| Wang YR, 2009 | 2003/01~2008/01 | Fuxin | Retrospective | 45 | Swab | 42~79 | Inpatient | Aerobical | No |
| Liang CF, 2009 | 2004/06~2008/02 | Linyi | Retrospective | 59 | Swab | 67.1 | Inpatient | Aerobical | Yes |
| Yang CZ, 2010 | 2008/07~2010/02 | Beijing | Retrospective | 116 | Swab | 63.7 | Inpatient | Aerobical | Yes |
| Yu HF, 2010 | 2009/01~2009/06 | Shanghai | Retrospective | 544 | Swab | 50~82 | Inpatient | Aerobical | Yes |
| Hong SC, 2010 | 2005/01~2009/12 | Fuzhou | Retrospective | 125 | Swab | 71.7 | Inpatient | Aerobical | Yes |
| Wan YM, 2010 | 2005/01~2009/06 | Shiyan | Retrospective | 57 | Swab | 61.2 | Inpatient | Aerobical | Yes |
| Feng XQ, 2010 | 2008/01~2009/12 | Zhongshan | Retrospective | 73 | Swab, Tissue | 65.2 | Inpatient | Aerobical | Yes |
| Lu LQ, 2010 | 2004/01~2000/06 | Shanghai | Retrospective | 60 | Swab | 69.8 | Inpatient | Aerobical | Yes |
| Wang M, 2010 | 2006/01~2009/03 | Chengdu | Retrospective | 67 | Swab, Tissue | 35~82 | Inpatient | Aerobical | Yes |
| Li Q, 2010 | 2004/01~2008/12 | Gaozhou | Retrospective | 81 | Swab | 63 | Inpatient | Aerobical | Yes |
| Li JB, 2010 | 2003/01~2007/12 | Shenyang | Retrospective | 91 | Swab | N/A | Inpatient | Aerobical | Yes |
| Wang SH,2010 | 2004/01-2006/12 | JiangSu | Retrospective | 118 | Swab | 60.8 | Inpatient | Aerobical | No |
| Xie WQ, 2011 | 2006/01~2009/12 | Guigang | Retrospective | 118 | Swab | 42~79 | Inpatient | Aerobical | Yes |
| Feng XF, 2011 | 2008/08~2010/08 | Changzhou | Retrospective | 75 | Swab | N/A | Inpatient | Aerobical | Yes |
| Fang H, 2011 | 2006/01~2011/02 | Cangzhou | Retrospective | 713 | Swab, Tissue | 63.7 | Inpatient | Aerobical | Yes |
| Wang SG, 2011 | 2009/01~2010/12 | Fengxian | Retrospective | 48 | Swab | 66.7 | Inpatient | Aerobical | Yes |
| Hu CL, 2011 | 2001/01~2010/01 | Suzhou | Retrospective | 58 | Swab | 68.8 | Inpatient | Aerobical | Yes |
| Yang QY,2011 | 2007/01~2010/10 | Guangzhou | Retrospective | 94 | Swab | 63.9 | Inpatient | Aerobical | No |
| Zhang SH, 2011 | 2008/01~2010/06 | Jinan | Retrospective | 68 | Swab | 69.2 | Inpatient | Aerobical | Yes |
| Lv CL, 2011 | 2008/01~2010/11 | Xiangyang | Retrospective | 281 | Swab | N/A | N/A | Aerobical | Yes |
| Li YH, 2011 | 1996/01~2009/12 | Chengdu | Retrospective | 507 | Swab | N/A | N/A | Aerobical | Yes |
| Lin CJ, 2011 | 2004/01~2009/12 | Shantou | Retrospective | 216 | Swab, Tissue | 66.3 | Inpatient  & Outpatient | Aerobical | Yes |
| Zeng YQ, 2012 | 2009/01~2011/06 | Shiyan | Retrospective | 57 | Swab, Tissue | 61.2 | Inpatient | Aerobical | Yes |
| Guan XH, 2012 | 1997/01~2010/12 | Beijing | Retrospective | 379 | Swab, Tissue | N/A | Inpatient | Aerobical | Yes |
| Li LR, 2012 | 2009/01~2011/12 | Changchun | Retrospective | 62 | Swab, Tissue | 57.5 | Inpatient | Aerobical | Yes |
| Yang Y, 2012 | 2005/01~2011/03 | Chengdu | Retrospective | 125 | Swab | 57.4 | Inpatient | Aerobical | Yes |
| Liang LR, 2012 | 2006/01~2009/12 | Changchun | Retrospective | 95 | Swab, Tissue | 65.2 | Inpatient | Aerobical | Yes |
| Wu DD, 2012 | 2009/01~2010/12 | Shaoxing | Retrospective | 135 | Swab | N/A | Inpatient | Aerobical | Yes |
| Fan H, 2012 | 2006/01~2010/11 | Cangzhou | Retrospective | 357 | Swab, Tissue | 63.7 | Inpatient | Aerobical | Yes |
| Liu JF, 2012 | 2003/01~2011/12 | Guangzhou | Retrospective | 109 | Swab, Tissue | 70.5 | Inpatient | Aerobical | Yes |
| Huang DW, 2012 | 2006/09~2011/03 | Changsha | Retrospective | 34 | Swab, Tissue | 62.3 | Inpatient | Aerobical | Yes |
| Liu JF, 2012 | 2003/01~2010/12 | Guangzhou | Retrospective | 69 | Swab, Tissue | 70.8 | Inpatient | Aerobical | Yes |
| Wen ZG, 2012 | 2009/01~2010/12 | Jiangmen | Retrospective | 73 | Swab | 66.5 | Inpatient | Aerobical | Yes |
| Li HQ, 2012 | 2006/01~2010/12 | Nanjing | Retrospective | 75 | Swab | 67.1 | Inpatient | Aerobical | Yes |
| Xie YJ, 2012 | 2009/01~2011/09 | Guangzhou | Retrospective | 153 | Swab, Tissue | N/A | Inpatient | Aerobical | Yes |
| BO LH, 2012 | 2006/01~2009/12 | Baotou | Retrospective | 36 | Swab, Tissue | N/A | Inpatient | Aerobical | Yes |
| Ren LZ, 2012 | 2008/07~2011/06 | Yuyao | Retrospective | 148 | Swab | NA | Inpatient | Aerobical | Yes |
| Lei CQ, 2012 | 2007/09~2012/02 | Beijing | Retrospective | 74 | Swab | 67.3 | Inpatient | Aerobical | Yes |
| He YG, 2012 | 2010/01~2010/12 | Nanning | Retrospective | 86 | Swab, Tissue | 64 | Inpatient | Aerobical | No |
| Zhang Z, 2012 | 2006/01~2010/12 | Changsha | Retrospective | 284 | Swab | 60.7 | Inpatient | Aerobical | No |
| Lin HG, 2012 | 2009/11~2011/10 | Guangzhou | Retrospective | 49 | Swab | 51 | Inpatient | Aerobical | Yes |
| Ding Q, 2012 | 2008/01~2010/06 | Tianjin | Retrospective | 388 | Swab | 65 | Inpatient | Aerobical | No |
| Xu XH, 2012 | 2011/01~2012/06 | Chongqing | Retrospective | 65 | Swab | 72 | Inpatient | Aerobical | Yes |
| Hai J, 2013 | 2011/01~2012/10 | Weihui | Prospective | 116 | Swab | 65.3 | Inpatient | Aerobical | Yes |
| Han J, 2013 | 2010/01~2011/12 | Tianjin | Retrospective | 80 | Swab | 67.1 | Inpatient | Aerobical | Yes |
| Zhou Y, 2013 | 2009/01~2011/12 | Nanning | Retrospective | 108 | Swab | N/A | Inpatient | Aerobical | Yes |
| Mo ZW, 2013 | 2004/01~2012/08 | Haikou | Retrospective | 82 | Swab | 68.4 | Inpatient | Aerobical | Yes |
| Man A, 2013 | 2008/01~2012/12 | Nanning | Retrospective | 65 | Swab | 55.6 | Inpatient | Aerobical | Yes |
| Li XL, 2013 | 2010/01~2011/05 | Nanchong | Retrospective | 66 | Swab | 64.9 | Inpatient | Aerobical | Yes |
| Ma MK, 2013 | 2011/06~2012/12 | Tianjin | Retrospective | 230 | Swab | 65.6 | Inpatient  & Outpatient | Aerobical | Yes |
| Shen X, 2013 | 2011/06~2012/12 | Tianjin | Retrospective | 91 | Swab | 64.3 | Inpatient | Aerobical | Yes |
| Wang XW, 2013 | 2005/01~2011/12 | Xining | Retrospective | 173 | Swab, Tissue | N/A | Inpatient | Aerobical | Yes |
| Liang QH, 2013 | 2009/01~2013/01 | Nanning | Retrospective | 105 | Swab, Tissue | 69.4 | Inpatient | Aerobical | Yes |
| Liang B, 2013 | 2012/02~2013/02 | Quanzhou | Retrospective | 52 | Swab | 62 | Inpatient | Aerobical | Yes |
| Liao T, 2013 | 2007/08~2012/04 | Nanchong | Retrospective | 72 | Swab | 64.4 | Inpatient | Aerobical | Yes |
| Zhan DS, 2013 | 2010/01~2012/12 | Sanming | Retrospective | 65 | Swab | 63.3 | Inpatient | Aerobical | Yes |
| Zhong JX, 2013 | 2010/01~2012/04 | Wuzhou | Retrospective | 339 | Swab, Tissue | 44.1 | Inpatient | Aerobical | Yes |
| Deng TT, 2013 | 2009/01~2013/06 | Liuzhou | Retrospective | 178 | Swab, Tissue | 58.2 | Inpatient | Aerobical | No |
| Guo J, 2013 | 2008/07~2012/07 | Tianjin | Retrospective | 575 | Tissue | 64.4 | Inpatient | Aerobical | Yes |
| Zhang Q, 2013 | 2011/01~2012/06 | Nanchang | Retrospective | 84 | Swab | N/A | Inpatient | Aerobical | Yes |
| Feng SH, 2013 | 2009/06-2011/05 | Tianjin | Retrospective | 429 | Swab | 64.24 | Inpatient | Aerobical  & Anaerobic | No |
| Wu Y, 2014 | 2011/05~2011/11 | Foshan | Retrospective | 79 | Swab | 59 | Inpatient | Aerobical | Yes |
| Chen H, 2014 | 2010/02~2013/05 | Lanxi | Retrospective | 164 | Swab | 61.4 | Inpatient | Aerobical | Yes |
| Guan TY, 2014 | 2006/01~2011/12 | Tangshan | Retrospective | 834 | Swab, Tissue | N/A | Inpatient | Aerobical | Yes |
| Wu D, 2014 | 2009/01~2013/12 | Beijing | Retrospective | 937 | Swab, Tissue | N/A | Inpatient | Aerobical | Yes |
| Che LQ, 2014 | 2010/05~2013/05 | Qiqihaer | Retrospective | 160 | Swab, Tissue | N/A | Inpatient | Aerobical | Yes |
| Chen XW, 2014 | 2011/01~2013/01 | Hangzhou | Retrospective | 113 | Swab | 58.2 | Inpatient | Aerobical | Yes |
| Li L, 2014 | 2012/06~2013/06 | Qingdao | Retrospective | 137 | Swab, Tissue | N/A | Inpatient  & Outpatient | Aerobical | Yes |
| Zhao YH, 2014 | 2011/04~2013/06 | Fenghua | Retrospective | 160 | Swab, Tissue | N/A | Inpatient | Aerobical | Yes |
| Shao F, 2014 | 2010/04~2012/12 | Changsha | Retrospective | 71 | Swab, Tissue | 54.5 | Inpatient  & Outpatient | Aerobical | Yes |
| Liu YZ, 2014 | 2010/01~2013/10 | Zhenjiang | Retrospective | 83 | Swab | 57 | Inpatient | Aerobical | Yes |
| Wang XG, 2014 | 2010/06~2013/12 | Xian | Retrospective | 102 | Swab, Tissue | 57.8 | Inpatient | Aerobical | Yes |
| Ji XZ, 2014 | 2010/01~2012/12 | Lishui | Retrospective | 152 | Swab, Tissue | 63.5 | Inpatient | Aerobical | Yes |
| Zeng HQ, 2014 | 2012/05~2013/05 | Dongguan | Retrospective | 76 | Swab | 65.4 | Inpatient | Aerobical | Yes |
| Pan XQ, 2014 | 2012/01~2014/01 | Liujiang | Retrospective | 85 | Swab, Tissue | 62.5 | Inpatient | Aerobical | Yes |
| Zheng SY, 2014 | 2011/05~2013/05 | Huzhou | Prospective | 178 | Swab, Tissue | 64.2 | Inpatient | Aerobical | Yes |
| Shao LJ, 2014 | 2008/01~2013/10 | Yuyao | Prospective | 225 | Swab, Tissue | N/A | Inpatient | Aerobical | Yes |
| Ma ZX, 2014 | 2009/04~2013/06 | Hebei | Retrospective | 128 | Swab | 70.1 | Inpatient | Aerobical | No |
| Liang JC, 2014 | 2012/06~2013/06 | Chengdu | Prospective | 181 | Swab | 65.3 | Inpatient | Aerobical | Yes |
| Zhang B, 2014 | 2011/01~2013/01 | Xian | Prospective | 257 | Tissue | N/A | Inpatient | Aerobical | No |
| Wu YL, 2014 | 2011/05~2013/07 | Tianjin | Retrospective | 50 | Swab | N/A | Inpatient | Aerobical | Yes |
| Fu H, 2014 | 2012/01~2014/01 | Changchun | Retrospective | 90 | Swab | 62 | Inpatient | Aerobical | Yes |
| Fan LQ, 2014 | 2011/06~2013/06 | Gaotang | Retrospective | 460 | Swab | 66.3 | Inpatient | Aerobical | Yes |
| Wang MR, 2014 | 2009/01~2013/12 | Enshi | Retrospective | 82 | Swab, Tissue | 65.3 | Inpatient | Aerobical  & Anaerobic | Yes |
| Guo GF, 2014 | 2011/01~2013/06 | Dongguan | Retrospective | 96 | Swab | 58.5 | Inpatient | Aerobical | Yes |
| Shen QY, 2014 | 2010/01~2013/08 | Wenzhou | Retrospective | 519 | Swab | 67.7 | Inpatient | Aerobical | Yes |
| Ji X, 2014 | 2011/01-2012/01 | Tianjin | Retrospective | 157 | Swab | N/A | Inpatient | Aerobical | No |
| Yao L, 2015 | 2012/01~2014/12 | Wuhu | Prospective | 95 | Swab | 60.2 | Inpatient | Aerobical | Yes |
| Ye M, 2015 | 2012/01~2014/12 | Wuzhou | Retrospective | 130 | Swab, Tissue | 61.2 | Inpatient | Aerobical | Yes |
| Zhang H, 2015 | 2014/01~2014/06 | Dongguan | Prospective | 50 | Swab | 62.6 | Outpatient | Aerobical | Yes |
| Chen Y, 2015 | 2011/01~2014/09 | Maanshan | Prospective | 125 | Swab | 60.2 | Inpatient | Aerobical | Yes |
| Wang B, 2015 | 2013/06~2014/06 | Shenyang | Retrospective | 38 | Swab | 58 | Inpatient | Aerobical | Yes |
| Zhou D, 2015 | 2013/01~2014/07 | Wuxi | Prospective | 110 | Swab | 61.3 | Inpatient | Aerobical | Yes |
| Liu Y, 2015 | 2014/05~2015/05 | Tangshan | Prospective | 108 | Swab | 61.3 | Inpatient | Aerobical | Yes |
| Fu H, 2015 | 2012/01~2014/01 | Changchun | Retrospective | 90 | Swab, Tissue | 62 | Inpatient | Aerobical | Yes |
| Chen YY, 2015 | 2012/01~2014/12 | Nangning | Retrospective | 111 | Swab | N/A | Inpatient | Aerobical | Yes |
| Xiao JM, 2015 | 2014/01~2015/01 | Zhongshan | Prospective | 115 | Swab, Tissue | 58 | Inpatient | Aerobical | No |
| Zhang D, 2015 | 2014/02~2014/12 | Beijing | Prospective | 26 | Bone | 58.3 | Inpatient | Aerobical | No |
| Rao XP, 2015 | 2012/01~2014/12 | Qingdao | Retrospective | 210 | Swab, Tissue | 62.3 | Inpatient | Aerobical | Yes |
| Ping XD, 2015 | 2011/01~2012/06 | Huludao | Retrospective | 460 | Swab | 66.3 | Inpatient | Aerobical | Yes |
| Huang Y, 2015 | 2011/01~2014/12 | Guangzhou | Retrospective | 372 | Swab, Tissue, Bone | 61.9 | Inpatient | Aerobical | Yes |
| Chang X, 2015 | 2013/01~2013/12 | Shanghai | Prospective | 200 | Swab | 67 | Inpatient | Aerobical | Yes |
| Wang G, 2015 | 2004/11-2011/12 | Hebei | Retrospective | 423 | Swab | 68.76 | Inpatient | Aerobical  & Anaerobic | No |
| Zhong YF, 2016 | 2013/01~2015/12 | Lishui | Prospective | 115 | Swab | 67.2 | Inpatient | Aerobical | Yes |
| Lou BC, 2016 | 2013/01~2015/10 | Jinhua | Prospective | 108 | Swab | 61.8 | Inpatient | Aerobical | Yes |
| Zhao L, 2016 | 2014/01~2016/01 | Chongqing | Retrospective | 216 | Swab, Tissue | 63.5 | Inpatient | Aerobical | Yes |
| He LJ, 2016 | 2014/01~2015/06 | Dongguan | Retrospective | 74 | Swab | 58.4 | Inpatient | Aerobical | Yes |
| Chen F, 2016 | 2010/01~2015/05 | Hefei | Retrospective | 89 | Swab | 61.5 | Inpatient | Aerobical | Yes |
| Lin LP, 2016 | 2013/01~2015/08 | Xiamen | Retrospective | 56 | Swab | 66 | Inpatient | Aerobical | Yes |
| Chi HY, 2016 | 2013/01~2015/01 | Weihai | Retrospective | 201 | Swab | 56 | Inpatient  & Outpatient | Aerobical | Yes |
| Guo DM, 2016 | 2014/01~2015/12 | Daqing | Retrospective | 379 | Swab, Tissue | 61 | Inpatient | Aerobical | Yes |
| Qiu P, 2016 | 2012/07~2014/07 | Chengdu | Retrospective | 88 | Swab, Tissue | 64.4 | Inpatient | Aerobical | Yes |
| Liu YF, 2016 | 2014/06~2015/06 | Hangzhou | Prospective | 120 | Swab | 63.2 | Inpatient | Aerobical | Yes |
| Zhang WW, 2016 | 2013/12~2015/12 | Qingdao | Prospective | 120 | Swab | 66.6 | Inpatient | Aerobical | Yes |
| Dai J, 2016 | 2013/09~2015/08 | Wuhan | Retrospective | 180 | Swab | 64 | Inpatient | Aerobical | Yes |
| Ma YN, 2016 | 2014/06~2015/06 | Shijiazhuang | Prospective | 320 | Swab | 67.2 | Inpatient | Aerobical | Yes |
| Diao BB, 2016 | 2016/01~2016/06 | Changchun | Retrospective | 35 | Swab, Tissue | 58.1 | Inpatient | Aerobical | Yes |
| Liang JY, 2016 | 2012/01~2015/12 | Guangzhou | Retrospective | 292 | Swab, Tissue | 63 | Inpatient | Aerobical | Yes |
| Wang Y, 2016 | 2014/01~2016/01 | Weihai | Retrospective | 157 | Swab, Tissue | 58.6 | Inpatient | Aerobical  & Anaerobic | No |
| Wang XJ, 2016 | 2009/03~2016/03 | Chongqing | Retrospective | 133 | Tissue | N/A | Inpatient | Aerobical | Yes |
| Xu B, 2016 | 2012/01~2014/12 | Zhangjiakou | Retrospective | 705 | Swab, Tissue, Bone | 59.5 | Inpatient | Aerobical | Yes |
| Huang Y, 2016 | 2014/10-2015/07 | Guangzhou | Retrospective | 56 | Swab | N/A | Inpatient | Aerobical  & Anaerobic | No |
| Li Z, 2017 | 2015/06~2016/08 | Danzhou | Prospective | 88 | Swab, Tissue | 64.4 | Inpatient | Aerobical | Yes |
| Xie GY, 2017 | 2015/03~2016/07 | Xingyi | Prospective | 120 | Swab | 43.6 | Inpatient | Aerobical | Yes |
| Le ZH, 2017 | 2011/02~2013/12 | Jiaozuo | Prospective | 92 | Swab | 61.3 | Inpatient | Aerobical | Yes |
| Jiang H, 2017 | 2013/06~2016/12 | Xinyi | Retrospective | 56 | Swab | 56.2 | Inpatient | Aerobical | Yes |
| Wang LP, 2017 | 2012/01~2016/01 | Fuzhou | Retrospective | 387 | Swab, Tissue | 69.5 | Inpatient | Aerobical | Yes |
| Yu P, 2017 | 2013/01~2016/12 | Tianjin | Prospective | 103 | Swab | 61.7 | Inpatient | Aerobical | Yes |
| Zhao YB, 2017 | 2013/01~2015/12 | Taiyuan | Retrospective | 80 | Swab, Tissue | 68 | Inpatient | Aerobical | Yes |
| Pan JJ, 2017 | 2014/01~2015/12 | Dongguan | Retrospective | 620 | Swab | 63 | Inpatient | Aerobical | Yes |
| Zhou FY, 2017 | 2013/05~2016/07 | Macheng | Retrospective | 122 | Swab | 53.3 | Inpatient | Aerobical | Yes |
| Li LY, 2017 | 2014/09~2016/09 | Weinan | Retrospective | 100 | Swab, Tissue | 65.4 | Inpatient | Aerobical | Yes |
| He XY, 2017 | 2014/01~2016/12 | Foshan | Retrospective | 93 | Swab, Tissue | 65.2 | Inpatient | Aerobical | Yes |
| Zhou XF, 2017 | 2010/01~2014/12 | Beijing | Retrospective | 1201 | Swab, Tissue | 59.2 | Inpatient | Aerobical | Yes |
| Zhao WX, 2017 | 2015/01~2016/12 | Guangzhou | Retrospective | 201 | Swab, Tissue | 60.5 | Inpatient | Aerobical | Yes |
| Wei W, 2017^#^ | 2006/07~2016/06 | Zhengzhou | Retrospective | 287 | Swab | 60.8 | Inpatient | Aerobical | Yes |
| Chen H, 2017 | 2015/11~2017/07 | Linyi | Prospective | 70 | Swab | 58.2 | Inpatient | Aerobical | Yes |
| Chen C, 2017 | 2013/01~2016/12 | Foshan | Retrospective | 68 | Swab | 63.1 | Inpatient | Aerobical | No |
| Zhang BH, 2017 | 2002/02~2016/12 | Dalian | Retrospective | 124 | Swab | 74 | Inpatient | Aerobical | Yes |
| Lin H, 2017 | 2012/02~2014/05 | Zhuhai | Retrospective | 38 | Swab | 65 | Inpatient | Aerobical | No |
| Xu M, 2017 | 2011/01~2015/12 | Zhumadian | Retrospective | 425 | N/A | 59.6 | Inpatient | Aerobical | Yes |
| Yang WX, 2017 | 2014/01~2016/10 | Taian | Retrospective | 160 | Swab | N/A | Inpatient | Aerobical | Yes |
| Wu WX, 2017 | 2009/01-2014/12 | Guangzhou | Retrospective | 312 | Swab | N/A | Inpatient | Aerobical | No |
| Wen B, 2018 | 2010/01~2017/12 | Beijing | Retrospective | 304 | Swab | N/A | Inpatient  & Outpatient | Aerobical | Yes |
| Zhan GG, 2018 | 2010/06~2017/03 | Shijiazhuang | Retrospective | 114 | Swab | N/A | Inpatient | Aerobical | Yes |
| Xu XJ, 2018 | 2016/08~2018/04 | Wuxi | Retrospective | 120 | Swab | 65.4 | Inpatient | Aerobical | Yes |
| Liu JL, 2018 | 2016/01~2017/12 | Wuhan | Retrospective | 54 | swab | N/A | Inpatient | Aerobical | Yes |
| Zhang LQ, 2018 | 2013/04~2017/04 | Luxi | Retrospective | 69 | swab | 59 | Inpatient | Aerobical | Yes |
| Wang GH, 2018 | 2013/01~2017/01 | Shenyang | Retrospective | 116 | swab | 70.9 | Inpatient | Aerobical | Yes |
| Hu JM, 2018 | 2015/01~2017/08 | Chengdu | Retrospective | 140 | Swab | 60.5 | Inpatient | Aerobical | Yes |
| Li XL, 2018 | 2011/01~2016/09 | Shihezi | Retrospective | 135 | swab | 60.21 | Inpatient | Aerobical | No |
| Cheng LH, 2018 | 2016/01~2016/12 | Changsha | Retrospective | 119 | swab | 55.27 | Inpatient | Aerobical | Yes |
| Wang XX, 2018 | 2014/01~2017/06 | Tianjin | Retrospective | 103 | swab | 62 | Inpatient | Aerobical | Yes |
| Wang XF, 2018 | 2014/01~2017/06 | Ningbo | Retrospective | 125 | swab | 61.1 | Inpatient | Aerobical | No |
| Zhou LL, 2018 | 2014/01~2015/12 | Wenzhou | Retrospective | 121 | swab | N/A | Inpatient | Aerobical | Yes |
| Li L, 2018 | 2015/12~2017/11 | Changsha | Retrospective | 85 | swab | 62.3 | Inpatient | Aerobical | Yes |
| Liu YB, 2018 | 2017/01~2017/12 | Beijing | Retrospective | 75 | swab | 67.5 | Inpatient | Aerobical | Yes |
| Wang YH, 2018 | 2015/06~2017/06 | Wuhan | Retrospective | 122 | swab | 67.49 | Inpatient | Aerobical | No |
| Xiao GB, 2018 | 2011/10~2016/12 | Ziyan | Retrospective | 116 | swab | 56.12 | Inpatient | Aerobical | Yes |
| Wang C, 2018 | 2016/01~2016/07 | Sanya | Retrospective | 104 | swab | N/A | Inpatient | Aerobical | Yes |
| Qi JZ, 2018 | 2013/09~2017/11 | Xuchang | Retrospective | 100 | swab | N/A | Inpatient | Aerobical | Yes |
| Du M, 2018 | 2014/01~2016/12 | Wuhan | Retrospective | 103 | swab | 69.22 | Inpatient | Aerobical | No |
| Li M, 2018 | 2016/06~2017/02 | Xian | Retrospective | 193 | swab | N/A | Inpatient | Aerobical | Yes |
| Zhao L, 2018 | 2014/01~2016/01 | Chongqing | Retrospective | 216 | swab | N/A | Inpatient | Aerobical | Yes |
| Mou XQ, 2018 | 2017/03~2018/09 | Daqing | Retrospective | 62 | swab | 59.2 | Inpatient | Aerobical | Yes |
| Yan XX, 2018 | 2016/08~2018/10 | Daqing | Retrospective | 84 | swab | 58.3 | Inpatient | Aerobical | Yes |
| Yang L, 2018 | 2014/01~2015/12 | Qingdao | Retrospective | 125 | swab | 71.71 | Inpatient | Aerobical | Yes |
| Li X, 2018 | 2010/01-2014/12 | Beijin | Retrospective | 456 | Swab | 64.7 | Inpatient | Aerobical  & Anaerobic | No |
| Zhang XF, 2019 | 2014/01~2018/12 | Beijing | Retrospective | 115 | swab | 62.58 | Inpatient | Aerobical | No |
| Li H, 2019 | 2016/08~2019/06 | Changchun | Retrospective | 78 | swab | 59.2 | Inpatient | Aerobical | Yes |
| Mu Q, 2019 | 2017/03~2018/02 | Nanjin | Retrospective | 157 | swab | 64.1 | Inpatient | Aerobical | Yes |
| Li YM, 2019 | 2016/01~2018/04 | Huizhou | Retrospective | 80 | swab | 58.3 | Inpatient | Aerobical | Yes |
| Wang Y, 2019 | 2016/05~2018/05 | Wulumuqi | Retrospective | 316 | swab | 63.4 | Inpatient | Aerobical | Yes |
| Li HY, 2019 | 2015/01~2017/01 | Shanggao | Retrospective | 42 | swab | 58.6 | Inpatient | Aerobical | Yes |
| Wang XF, 2019 | 2016/01~2018/12 | Chongqin | Retrospective | 160 | swab | 64.86 | Inpatient | Aerobical | Yes |
| Zhao P, 2019 | 2014/01~2016/12 | Beijin | Retrospective | 563 | swab | N/A | Inpatient | Aerobical | Yes |
| Xie SY, 2019 | 2016/07~2018/07 | Hefei | Retrospective | 52 | swab | 50 | Inpatient | Aerobical | Yes |
| Zhan TF, 2019 | 2016/06~2018/06 | Pulin | Retrospective | 180 | swab | 54.76 | Inpatient | Aerobical | Yes |
| Guan XF, 2019 | 2017/02~2019/02 | Jilin | Retrospective | 80 | swab | 66.35 | Inpatient | Aerobical | Yes |
| Liu JM, 2019 | 2016/11~2018/11 | Jinin | Retrospective | 74 | swab | 57.45 | Inpatient | Aerobical | Yes |
| Yang XJ, 2019 | 2018/01~2018/09 | Yunyi | Retrospective | 92 | swab | 60.4 | Inpatient | Aerobical | Yes |
| Zou WY, 2019 | 2018/06~2019/06 | Jinin | Retrospective | 180 | swab | 53.34 | Inpatient | Aerobical | Yes |
| Su T, 2019 | 2015/01~2018/01 | Jinin | Retrospective | 50 | tissue | 62.4 | Inpatient | Aerobical | Yes |
| Zhao C, 2019 | 2016/07~2018/07 | Zhenzhou | Retrospective | 230 | swab | 61.36 | Inpatient | Aerobical | No |
| Su JF, 2019 | 2015/01~2017/12 | Guangzhou | Retrospective | 147 | swab | N/A | Inpatient | Aerobical | Yes |
| Liu CL, 2019 | 2012/01~2017/12 | Kunming | Retrospective | 267 | swab | N/A | Inpatient | Aerobical | Yes |
| Wang HM, 2019 | 2016/06~2018/03 | Meizhou | Retrospective | 96 | swab | 58.42 | Inpatient | Aerobical | Yes |
| Yang XM, 2019 | 2016/04~2018/04 | Beijin | Retrospective | 150 | swab | 43.5 | Inpatient | Aerobical | Yes |
| Shi AL, 2019 | 2017/02~2019/02 | Xinfeng | Retrospective | 62 | swab | 66.36 | Inpatient | Aerobical | Yes |
| Shi W, 2019 | 2018/02~2019/01 | Huizhou | Retrospective | 118 | swab | N/A | Inpatient | Aerobical | Yes |
| Yang ZH, 2019 | 2017/01~2018/12 | Wuhu | Retrospective | 133 | swab | N/A | Inpatient | Aerobical | Yes |
| Liu HY, 2019 | 2018/03~2019/02 | Fengchen | Retrospective | 60 | swab | 58.02 | Inpatient | Aerobical | Yes |
| Wang S, 2019 | 2016/01~2018/09 | Wuhu | Retrospective | 52 | swab | 59.90 | Inpatient | Aerobical | Yes |
| Pang JR, 2020 | 2013/01~2018/12 | Lingbo | Retrospective | 144 | swab | 62.49 | Inpatient | Aerobical | Yes |
| Li SM, 2020 | 2018/05~2019/05 | Xinxiang | Retrospective | 72 | swab | 48.98 | Inpatient | Aerobical | Yes |
| Cheng Z, 2020 | 2018/01~2018/12 | Luoyang | Retrospective | 332 | swab | N/A | Inpatient | Aerobical | Yes |
| Xie SY, 2020 | 2016/07~2018/07 | Hefei | Retrospective | 184 | swab | 50 | Inpatient | Aerobical | Yes |
| LiaoTT, 2020 | 2016/01~2018/12 | Chengdu | Retrospective | 79 | swab | N/A | Inpatient | Aerobical | Yes |
| Yan CY, 2020 | 2018/01~2019/02 | Wenzhou | Retrospective | 198 | swab | 62.36 | Inpatient | Aerobical | Yes |
| Fu QR, 2020 | 2015/03~2020/03 | Guangzhou | Retrospective | 102 | swab | 62.2 | Inpatient | Aerobical | Yes |
| Wang Y, 2020 | 2018/01~2019/05 | Jinan | Retrospective | 86 | swab | 58.42 | Inpatient | Aerobical | No |
| Li YF, 2020 | 2017/05~2019/04 | Wugang | Retrospective | 135 | swab | 63.29 | Inpatient | Aerobical | Yes |
| Peng F, 2020 | 2018/05~2019/04 | Lin Y | Retrospective | 63 | swab | 62.25 | Inpatient | Aerobical | No |
| Li M, 2020 | 2017/09~2019/03 | Zhengzhou | Retrospective | 60 | swab, Tissue, Bone | 60.28 | Inpatient | Aerobical | Yes |
| Hu ZP, 2020 | 2016/09~2019/09 | Enshi | Retrospective | 128 | Swab | 66.89 | Inpatient | Aerobical | Yes |
| Liu YM, 2020 | 2017/02~2019/06 | Nanyang | Retrospective | 148 | swab | 68.4 | Inpatient | Aerobical | Yes |
| Jiang YD,2 020 | 2013/01~2018/12 | Ninbo | Retrospective | 88 | swab | N/A | Inpatient | Aerobical | Yes |
| Xiao Y, 2020 | 2015/12~2019/01 | Weihai | Retrospective | 200 | swab | 46.47 | Inpatient | Aerobical | Yes |
| Wang LL, 2020 | 2017/01~2018/12 | Beijin | Retrospective | 246 | swab | N/A | Inpatient | Aerobical | Yes |
| Yang RM, 2020 | 2013/01~2019/07 | Chongqin | Retrospective | 286 | swab | N/A | Inpatient | Aerobical | Yes |
| Li M, 2020 | 2017/09~2020/01 | Zhengzhou | Retrospective | 105 | Swab, Tissue, Bone | 60.16 | Inpatient | Aerobical | Yes |

Table S2. Pooled rates of microorganisms in eastern and western parts of China during the entire 20-year period.

| Microorganisms | Eastern | Western | Total |
| --- | --- | --- | --- |
| Patients | 32958 | 5786 | 38744 |
| *Aerobic gram-positives*  *Staphylococcus aureus* | 6774(19.21) | 1232(19.98) | 8006 |
| *MRSA* | 578(8.53) | 82(6.66) | 660(8.24) |
| *Enterococcus* spp. | 3230(9.16) | 585(9.49) | 3815 |
| *CoN Staphylococcus* | 3594(10.19) | 626(10.15) | 4220 |
| *Streptococcus* spp. | 1283(3.64) | 236(3.83) | 1519 |
| *Other gram-positives* | 1427(4.05) | 310(5.03) | 1737 |
| *Total gram-positives* | 16308(46.25) | 2989(48.47) | 19297 |
| *Escherichia coli* | 3585(10.17) | 675(10.95) | 4260 |
| *Klebsiella* spp. | 1804(5.12) | 340(5.51) | 2144 |
| *Proteus* spp. | 2305(6.54) | 449(7.28) | 2754 |
| *Enterobacter* spp. | 1501(4.26) | 321(5.21) | 1822 |
| *P.aeruginosa.* | 3716(10.54) | 485(7.86) | 4201 |
| *Acinetobacter* spp. | 963(2.73) | 102(1.65) | 1065 |
| *Other Gram-negatives* | 2803(7.95) | 496(8.04) | 3299 |
| *Total gram-negative* | 16677(47.30) | 2868(46.51) | 19545 |
| *Obligate anaerobes* | 645(1.83) | 0(0) | 645 |
| *Fungus* | 1630(4.62) | 310(5.03) | 1940 |
| Total | 35260 | 6167 | 41427 |

Table S3. Pooled rates of microorganisms in northern and southern parts of China during the entire 20-year period.

| Microorganisms | Southern | Northern | Total |
| --- | --- | --- | --- |
| Patients | 17073 | 21671 | 38744 |
| *Aerobic gram-positives*  *Staphylococcus aureus* | 3582(19.25) | 4424(19.39) | 8006 |
| *MRSA* | 251(7.00) | 409(9.25) | 660 |
| *Enterococcus* spp. | 1953(10.50) | 1862(8.16) | 3815 |
| *CoN Staphylococcus* | 1973(10.60) | 2247(9.85) | 4220 |
| *Streptococcus* spp. | 756(4.06) | 763(3.34) | 1519 |
| *Other gram-positives* | 683(3.67) | 1054(4.62) | 1737 |
| *Total gram-positives* | 8947(48.08) | 10350(45.35) | 19297 |
| *Escherichia coli* | 2094(11.25) | 2166(9.49) | 4260 |
| *Klebsiella* spp. | 953(5.12) | 1191(5.22) | 2144 |
| *Proteus* spp. | 1389(7.46) | 1365(5.98) | 2754 |
| *Enterobacter* spp. | 805(4.33) | 1017(4.46) | 1822 |
| *P.aeruginosa.* | 1658(8.91) | 2543(11.14) | 4201 |
| *Acinetobacter* spp. | 313(1.68) | 752(3.30) | 1065 |
| *Other Gram-negatives* | 1390(7.47) | 1909(8.37) | 3299 |
| *Total gram-negative* | 8602(46.23) | 10943(47.95) | 19545 |
| *Obligate anaerobes* | 72(0.39) | 573(2.51) | 645 |
| *Fungus* | 976(5.25) | 964(4.22) | 1940 |
| Total | 18607 | 22820 | 41427 |

Table S4. Pooled rates of microorganisms in four major geographical regions of China during the entire 20-year period.

| Microorganisms | South | North | Tibetan Plateau | [Northwest](C:/Users/Pro7/AppData/Local/Youdao/Dict/Application/5.1.36.3166/resultui/app:ds:northwest) | Total |
| --- | --- | --- | --- | --- | --- |
| Patients | 17073 | 20921 | 173 | 577 | 38744 |
| *Aerobic gram-positives*  *Staphylococcus aureus* | 3582(19.26) | 4247(19.22) | 28(14.07) | 149(27.65) | 8006 |
| *MRSA* | 251(7.01) | 367(8.64) | 0(0) | 42(28.19) | 660 |
| *Enterococcus* spp. | 1953(10.50) | 1791(8.11) | 34(17.09) | 37(6.86) | 3815 |
| *CoN Staphylococcus* | 1973(10.61) | 2154(9.75) | 29(14.57) | 64(11.87) | 4220 |
| *Streptococcus* spp. | 756(4.07) | 757(3.43) | 0(0) | 6(1.11) | 1519 |
| *Other gram-positives* | 683(3.67) | 1008(4.56) | 11(5.53) | 35(6.49) | 1737 |
| *Total gram-positives* | 8947(48.11) | 9957(45.07) | 102(51.26) | 291(53.99) | 19297 |
| *Escherichia coli* | 2094(11.26) | 2080(9.42) | 25(12.56) | 61(11.32) | 4260 |
| *Klebsiella* spp. | 953(5.12) | 1156(5.23) | 8(4.02) | 27(5.01) | 2144 |
| *Proteus* spp. | 1389(7.47) | 1295(5.86) | 19(9.55) | 51(9.46) | 2754 |
| *Enterobacter* spp. | 805(4.33) | 993(4.49) | 6(3.02) | 18(3.34) | 1822 |
| *P.aeruginosa.* | 1658(8.92) | 2505(11.34) | 5(2.51) | 33(6.12) | 4201 |
| *Acinetobacter* spp. | 313(1.68) | 736(3.33) | 8(4.02) | 8(1.48) | 1065 |
| *Other Gram-negatives* | 1390(7.47) | 1841(8.33) | 26(13.07) | 42(7.79) | 3299 |
| *Total gram-negative* | 8602(46.25) | 10606(48.01) | 97(48.74) | 240(44.53) | 19545 |
| *Obligate anaerobes* | 72(0.39) | 573(2.59) | 0(0) | 0(0) | 645 |
| *Fungus* | 976(5.25) | 956(4.33) | 0(0) | 8(1.48) | 1940 |
| Total | 18597 | 22092 | 199 | 539 | 41427 |

Table S5. Pooled rates of microorganisms in seven major geographical regions of China during the entire 20-year period.

| Microorganisms | [Northeast](C:/Users/Pro7/AppData/Local/Youdao/Dict/Application/5.1.36.3166/resultui/app:ds:northeast) | [North](C:/Users/Pro7/AppData/Local/Youdao/Dict/Application/5.1.36.3166/resultui/app:ds:North) [China](C:/Users/Pro7/AppData/Local/Youdao/Dict/Application/5.1.36.3166/resultui/app:ds:China) | East China | South China | [Central](C:/Users/Pro7/AppData/Local/Youdao/Dict/Application/5.1.36.3166/resultui/app:ds:Central) [China](C:/Users/Pro7/AppData/Local/Youdao/Dict/Application/5.1.36.3166/resultui/app:ds:China) | [Southwest](C:/Users/Pro7/AppData/Local/Youdao/Dict/Application/5.1.36.3166/resultui/app:ds:southwest) | [Northwest](C:/Users/Pro7/AppData/Local/Youdao/Dict/Application/5.1.36.3166/resultui/app:ds:northwest) | Total |
| --- | --- | --- | --- | --- | --- | --- | --- | --- |
| Patients | 2257 | 10051 | 10833 | 6849 | 4415 | 1366 | 2973 | 38744 |
| *Aerobic gram-positives*  *Staphylococcus aureus* | 518(18.99) | 2010(18.79) | 2172(20.00) | 1511(19.33) | 868(18.62) | 325(23.16) | 602(18.48) | 8006 |
| *MRSA* | 29(5.60) | 252(12.54) | 66(3.04) | 66(4.37) | 165(19.01) | 42(12.92) | 40(6.64) | 660 |
| *Enterococcus* spp. | 219(8.03) | 915(8.55) | 984(9.06) | 808(10.34) | 458(9.82) | 106(7.56) | 325(9.98) | 3815 |
| *CoN Staphylococcus* | 221(8.10) | 1080(10.10) | 1093(10.06) | 949(12.14) | 455(9.76) | 165(11.76) | 257(7.89) | 4220 |
| *Streptococcus* spp. | 122(4.47) | 292(2.73) | 438(4.03) | 318(4.07) | 143(3.07) | 40(2.85) | 166(5.10) | 1519 |
| *Other gram-positives* | 209(7.66) | 529(4.94) | 367(3.38) | 210(2.69) | 153(3.28) | 72(5.13) | 197(6.05) | 1737 |
| *Total gram-positives* | 1289(47.25) | 4826(45.11) | 5054(46.53) | 3796(48.55) | 2077(44.55) | 708(50.46) | 1547(47.50) | 19297 |
| *Escherichia coli* | 270(9.90) | 801(7.49) | 1249(11.50) | 1006(12.87) | 508(10.90) | 138(9.84) | 288(8.84) | 4260 |
| *Klebsiella* spp. | 185(6.78) | 396(3.70) | 637(5.87) | 427(5.46) | 255(4.83) | 57(4.06) | 187(5.74) | 2144 |
| *Proteus* spp. | 156(5.72) | 536(5.01) | 780(7.18) | 512(6.55) | 409(8.77) | 95(6.77) | 266(8.17) | 2754 |
| *Enterobacter* spp. | 157(5.76) | 488(4.56) | 333(3.07) | 349(4.46) | 229(4.91) | 111(7.91) | 155(4.76) | 1822 |
| *P.aeruginosa.* | 241(8.83) | 1301(12.16) | 1132(10.42) | 725(9.27) | 445(9.55) | 124(8.84) | 233(7.15) | 4201 |
| *Acinetobacter* spp. | 76(2.79) | 492(4.60) | 225(2.07) | 71(0.91) | 113(2.42) | 24(1.71) | 64(1.96) | 1065 |
| *Other Gram-negatives* | 223(8.17) | 925(8.65) | 861(7.93) | 505(6.46) | 349(7.49) | 113(8.05) | 323(9.92) | 3299 |
| *Total gram-negative* | 1308(47.95) | 4939(46.17) | 5217(48.03) | 3595(45.98) | 2308(49.51) | 662(47.18) | 1516(46.55) | 19545 |
| *Obligate anaerobes* | 0(0) | 538(5.03) | 42(0.39) | 2(0.03) | 63(1.35) | 0(0) | 0(0) | 645 |
| *Fungus* | 131(4.80) | 395(3.69) | 548(5.05) | 425(5.44) | 214(4.59) | 33(2.35) | 194(5.96) | 1940 |
| Total | 2728 | 10698 | 10861 | 7818 | 4662 | 1403 | 3257 | 41427 |

Table S6. Pooled rates of microorganisms in Asia from 2001 to 2020.

| Microorganisms | South-Eastern | Western | South Central | Eastern  inc. China | China | Asia |
| --- | --- | --- | --- | --- | --- | --- |
| Patients  (Number of articles) | 1311(6) | 1994(18) | 5184(44) | 39752(247) | 38744(245) | 48241(315) |
| *Aerobic gram-positives*  *Staphylococcus aureus* | 298(15.62) | 501(18.64) | 1456(20.74) | 8308(19.53) | 8006 (19.33) | 10563(19.50) |
| *MRSA* | 129(43.29) | 127(25.35) | 280(19.23) | 818(9.85) | 660(8.24) | 1354(12.82) |
| *Enterococcus* spp. | 86(4.51) | 220(8.18) | 525(7.48) | 3961(9.31) | 3815(9.21) | 4792(8.85) |
| *CoN Staphylococcus* | 27(1.42) | 146(5.43) | 316(4.50) | 4234(9.95) | 4220(10.19) | 4723(8.72) |
| *Streptococcus* spp. | 151(7.91) | 189(7.03) | 197(2.81) | 1604(3.77) | 1519(3.67) | 2141(3.95) |
| *Other gram-positives* | 22(1.15) | 28(1.04) | 187(2.66) | 1820(4.28) | 1737(4.19) | 2057(3.80) |
| *Total gram-positives* | 584(30.61) | 1084(40.33) | 2681(38.19) | 19927(46.84) | 19297 (46.58) | 24276(44.82) |
| *Escherichia coli* | 140(7.34) | 245(9.11) | 1105(15.74) | 4341(10.20) | 4260(10.28) | 5831(10.77) |
| *Klebsiella* spp. | 212(11.11) | 124(4.61) | 759(10.81) | 2184(5.13) | 2144(5.18) | 3279(6.05) |
| *Proteus* spp. | 207(10.85) | 194(7.22) | 741(10.56) | 2790(6.56) | 2754(6.65) | 3932(7.26) |
| *Enterobacter* spp. | 131(6.87) | 103(3.83) | 72(1.03) | 1832(4.31) | 1822(4.40) | 2138(3.95) |
| *P.aeruginosa.* | 314(16.46) | 387(14.40) | 996(14.19) | 4305(10.12) | 4201(10.14) | 6002(11.08) |
| *Acinetobacter* spp. | 50(2.62) | 49(1.82) | 182(2.59) | 1084(2.55) | 1065(2.57) | 1365(2.52) |
| *Other Gram-negatives* | 116(6.08) | 271(10.08) | 322(4.59) | 3476(8.17) | 3299(7.96) | 4185(7.73) |
| *Total gram-negative* | 1170(61.32) | 1373(51.08) | 4177(59.50) | 20012(47.04) | 19545 (47.18) | 26732(49.36) |
| *Obligate anaerobes* | 145(7.60) | 187(6.96) | 117(1.67) | 657(1.54) | 645(1.56) | 1106(2.04) |
| *Fungus* | 9(0.47) | 44(1.63) | 45(0.64) | 1949(4.58) | 1940(4.68) | 2047(3.78) |
| Total | 1908 | 2688 | 7020 | 42545 | 41427 | *54161* |

Table S7. Pooled rates of microorganisms in the world assessed 2001～2020.

| Microorganisms | Africa | America | Europe | Asia  Inc. China | World Area |
| --- | --- | --- | --- | --- | --- |
| Patients  (Number of articles) | 1170(6) | 3798(17) | 3383(21) | 48241(315) | 56,592(359) |
| *Aerobic gram-positives*  *Staphylococcus aureus* | 368(21.33) | 1401(19.48) | 1249(30.67) | 10563(19.50) | 13581(20.22) |
| *MRSA* | 99(26.90) | 182(12.99) | 288(23.06) | 1354(12.82) | 1923(14.16) |
| *Enterococcus* spp. | 150(8.70) | 908(12.62) | 392(9.63) | 4792(8.85) | 6242(9.30) |
| *CoN Staphylococcus* | 111(6.43) | 614(8.54) | 340(8.35) | 4723(8.72) | 5788(8.62) |
| *Streptococcus* spp. | 39(2.26) | 937(13.03) | 294(7.22) | 2141(3.95) | 3411(5.08) |
| *Other gram-positives* | 141(8.17) | 653(9.08) | 171(4.20) | 2057(3.80) | 3022(4.50) |
| *Total gram-positives* | 809(46.90) | 4513(62.74) | 2446(60.09) | 24276(44.82) | 32044(47.72) |
| *Escherichia coli* | 139(8.06) | 255(3.55) | 133(3.27) | 5831(10.77) | 6358(9.47) |
| *Klebsiella* spp. | 130(7.54) | 153(2.13) | 66(1.62) | 3279(6.05) | 3628(5.40) |
| *Proteus* spp. | 157(9.10) | 338(4.70) | 130(3.19) | 3932(7.26) | 4557(6.79) |
| *Enterobacter* spp. | 29(1.68) | 254(3.53) | 75(1.84) | 2138(3.95) | 2496(3.72) |
| *P.aeruginosa.* | 145(8.41) | 382(5.31) | 415(10.19) | 6002(11.08) | 6944(10.34) |
| *Acinetobacter* spp. | 33(1.91) | 109(1.52) | 57(1.40) | 1365(2.52) | 1564(2.33) |
| *Other Gram-negatives* | 64(3.71) | 475(6.60) | 573(14.07) | 4185(7.73) | 5297(7.89) |
| *Total gram-negative* | 697(40.41) | 1966(27.33) | 1449(35.58) | 26732(49.36) | 30844(45.93) |
| *Obligate anaerobes* | 197(11.42) | 654(9.09) | 95(2.33) | 1106(2.04) | 2052(3.06) |
| *Fungus* | 22(1.28) | 60(0.83) | 82(2.01) | 2047(3.78) | 2211(3.29) |
| Total | 1725 | 7193 | 4072 | *54161* | 67,151 |

Table S8. Percentage of in vitro susceptibility to selected antibiotic agents of Gram-positive aerobic isolates from diabetic foot infections in China.

| Antibiotic | *Staphylococcus aureus* | | | *Enterococcus* spp. | | | *Staphylococcus (coagulase negative)* | | | *Streptococcus* spp. | | |
| --- | --- | --- | --- | --- | --- | --- | --- | --- | --- | --- | --- | --- |
|  | N | T | % | N | T | % | N | T | % | N | T | % |
| Oxacillin | 1289 | 2330 | 55.32 | 95 | 512 | 18.55 | 286 | 873 | 32.76 | 65 | 117 | 55.56 |
| Methicillin | 32 | 86 | 37.21 | \ | \ | \ | \ | \ | \ | \ | \ | \ |
| Penicillin | 374 | 2576 | 14.52 | 793 | 1470 | 53.95 | 189 | 984 | 19.21 | 260 | 361 | 72.02 |
| Ampicillin | 207 | 1109 | 18.67 | 486 | 856 | 56.78 | 84 | 407 | 20.64 | 81 | 150 | 54.00 |
| Vancomycin | 3565 | 3638 | 97.99 | 1616 | 1754 | 92.13 | 1126 | 1172 | 96.08 | 344 | 357 | 96.36 |
| Teicoplanin | 1239 | 1293 | 95.82 | 434 | 499 | 86.97 | 413 | 457 | 90.37 | 44 | 62 | 70.97 |
| Linezolid | 1778 | 1826 | 97.37 | 801 | 910 | 88.02 | 513 | 553 | 92.77 | 159 | 199 | 79.90 |
| Ciprofloxacin | 1614 | 2685 | 60.11 | 726 | 1442 | 50.35 | 464 | 1038 | 44.70 | 125 | 210 | 59.52 |
| Levofloxacin | 1737 | 2678 | 64.86 | 777 | 1236 | 62.86 | 451 | 934 | 48.29 | 221 | 291 | 75.95 |
| Trimethoprim-sulfamethoxazole | 1216 | 1873 | 64.92 | 122 | 322 | 37.89 | 363 | 761 | 47.70 | 70 | 133 | 52.63 |
| Rifampicin | 1636 | 2080 | 78.65 | 342 | 694 | 49.28 | 504 | 647 | 77.90 | 35 | 50 | 70.00 |
| Fusidic acid | \ | \ | \ | \ | \ | \ | \ | \ | \ | \ | \ | \ |
| Ampicillin/sulbactam | 241 | 431 | 55.92 | 51 | 126 | 40.48 | 79 | 190 | 41.58 | 18 | 25 | 72.00 |
| Tetracycline | 1182 | 1907 | 61.98 | 266 | 821 | 32.40 | 399 | 719 | 55.49 | 85 | 186 | 45.70 |
| Erythromycin | 1088 | 2890 | 37.65 | 333 | 1284 | 25.93 | 307 | 926 | 33.15 | 125 | 291 | 42.96 |
| Clindamycin | 1264 | 2713 | 46.59 | 205 | 887 | 23.11 | 349 | 857 | 40.72 | 168 | 303 | 55.45 |

N: number of sensitive isolates, T: total number of isolates, %: percentage of sensitive isolates.

| Antibiotic | *Escherichia coli* | | | *Klebsiella* spp. | | | *Proteus* spp. | | | *Enterobacter* spp. | | | *P. aeruginosa* | | | *Acinetobacter* spp. | | |
| --- | --- | --- | --- | --- | --- | --- | --- | --- | --- | --- | --- | --- | --- | --- | --- | --- | --- | --- |
|  | N | T | % | N | T | % | N | T | % | N | T | % | N | T | % | N | T | % |
| Amikacin | 1128 | 1477 | 76.37 | 443 | 557 | 79.53 | 663 | 786 | 84.35 | 343 | 425 | 80.71 | 1021 | 1433 | 71.25 | 117 | 211 | 55.45 |
| Aztreonam | 456 | 921 | 49.51 | 260 | 387 | 67.18 | 444 | 528 | 84.09 | 231 | 344 | 67.15 | 579 | 971 | 59.63 | 44 | 94 | 46.81 |
| Cefepime | 545 | 1079 | 50.51 | 278 | 405 | 68.64 | 419 | 567 | 73.90 | 241 | 325 | 74.15 | 742 | 1157 | 64.13 | 53 | 102 | 51.96 |
| Cefoperazone-sulbactam | 523 | 704 | 74.29 | 259 | 329 | 78.72 | 207 | 258 | 80.23 | 121 | 171 | 70.76 | 575 | 1055 | 54.50 | 68 | 116 | 58.62 |
| Ceftazidime | 794 | 1512 | 52.51 | 444 | 660 | 67.27 | 675 | 853 | 79.13 | 238 | 421 | 56.53 | 919 | 1542 | 59.60 | 72 | 159 | 45.28 |
| Cefotaxime | 350 | 1440 | 24.31 | 158 | 307 | 51.47 | 315 | 484 | 65.08 | 51 | 125 | 40.80 | 345 | 809 | 42.65 | 37 | 89 | 41.57 |
| Ciprofloxacin | 463 | 1334 | 34.71 | 380 | 604 | 62.91 | 402 | 718 | 55.99 | 210 | 327 | 64.22 | 901 | 1436 | 62.74 | 79 | 161 | 49.07 |
| Gentamicin | 717 | 1521 | 47.14 | 427 | 674 | 63.35 | 464 | 760 | 61.05 | 245 | 405 | 60.49 | 865 | 1547 | 55.91 | 65 | 165 | 39.39 |
| Imipenem | 1430 | 1510 | 94.70 | 620 | 662 | 93.66 | 743 | 842 | 88.24 | 386 | 426 | 90.61 | 1300 | 1586 | 81.97 | 117 | 171 | 68.42 |
| Levofloxacin | 531 | 1314 | 40.41 | 353 | 492 | 71.75 | 465 | 660 | 70.45 | 183 | 322 | 56.83 | 737 | 1196 | 61.62 | 107 | 197 | 54.31 |
| Trimethoprim-sulfamethoxazole | 246 | 733 | 33.56 | 155 | 323 | 47.99 | 241 | 592 | 40.71 | 119 | 250 | 47.60 | 102 | 558 | 18.20 | 59 | 176 | 33.52 |
| Piperacillin/tazobactam | 817 | 1078 | 75.79 | 312 | 389 | 80.21 | 457 | 612 | 74.67 | 285 | 380 | 75.00 | 730 | 1065 | 68.54 | 83 | 188 | 44.15 |
| Ampicillin | 166 | 867 | 19.15 | 85 | 381 | 22.31 | 253 | 631 | 40.10 | 42 | 294 | 14.29 | 205 | 466 | 43.99 | 7 | 43 | 16.28 |
| Ampicillin-sulbactam | 227 | 489 | 46.42 | 129 | 214 | 60.28 | 239 | 381 | 62.73 | 71 | 127 | 55.91 | 90 | 204 | 44.12 | 71 | 115 | 61.74 |

Table S9. Percentage of in vitro susceptibility to selected antibiotic agents of Gram-negative aerobic isolates from diabetic foot infections in China.

N: number of sensitive isolates, T: total number of isolates, %: percentage of sensitive isolates.

Table S10. Percentage of in vitro susceptibility to selected antibiotic agents of Gram-positive aerobic isolates from diabetic foot infections in the world.

| Antibiotic | *Staphylococcus aureus* | | | *Enterococcus* spp. | | | *Staphylococcus (coagulase negative)* | | | *Streptococcus* spp. | | |
| --- | --- | --- | --- | --- | --- | --- | --- | --- | --- | --- | --- | --- |
|  | N | T | % | N | T | % | N | T | % | N | T | % |
| **Asia inc. China** |  |  |  |  |  |  |  |  |  |  |  |  |
| Oxacillin | 1317 | 2367 | 55.64 | 100 | 517 | 19.34 | \ | \ | \ | \ | \ | \ |
| Methicillin | 76 | 147 | 51.70 | \ | \ | \ | 7 | 16 | 43.75 | 4 | 5 | 80.00 |
| Penicillin | 416 | 2969 | 14.01 | 903 | 1653 | 54.63 | 193 | 1000 | 19.30 | 300 | 420 | 71.43 |
| Ampicillin | 385 | 1305 | 29.50 | 488 | 870 | 56.09 | \ | \ | \ | 86 | 155 | 55.48 |
| Vancomycin | 4019 | 4099 | 98.05 | 1806 | 1964 | 91.96 | 1162 | 1208 | 96.19 | 367 | 387 | 94.83 |
| Teicoplanin | 1247 | 1301 | 95.85 | 458 | 526 | 87.07 | 417 | 461 | 90.46 | \ | \ | \ |
| Linezolid | 1874 | 1932 | 97.00 | 888 | 1003 | 88.53 | 533 | 573 | 93.02 | \ | \ | \ |
| Ciprofloxacin | 1751 | 2929 | 59.78 | 792 | 1592 | 49.75 | 485 | 1080 | 44.91 | 157 | 244 | 64.34 |
| Levofloxacin | 1743 | 2694 | 64.70 | 799 | 1268 | 63.01 | \ | \ | \ | \ | \ | \ |
| Trimethoprim-sulfamethoxazole | 1392 | 2120 | 65.66 | 143 | 372 | 38.44 | 377 | 777 | 48.52 | 93 | 156 | 59.62 |
| Rifampicin | 1949 | 2418 | 80.60 | 342 | 708 | 48.31 | 520 | 669 | 77.73 | \ | \ | \ |
| Fusidic acid | 219 | 379 | 57.78 | 17 | 40 | 42.5 | \ | \ | \ | \ | \ | \ |
| Ampicillin/sulbactam | 241 | 465 | 51.83 | 62 | 175 | 35.43 | 81 | 194 | 41.75 | 18 | 43 | 41.86 |
| Tetracycline | 1363 | 2302 | 59.21 | 404 | 991 | 40.77 | 414 | 737 | 56.17 | 100 | 205 | 48.78 |
| Erythromycin | 1413 | 3443 | 41.04 | 435 | 1561 | 27.87 | 332 | 960 | 34.58 | 171 | 352 | 48.58 |
| Clindamycin | 1488 | 3091 | 48.14 | 238 | 952 | 25.00 | 369 | 883 | 41.79 | 190 | 331 | 57.40 |
|  | | | | | | | | | | | | |
| **Africa** |  |  |  |  |  |  |  |  |  |  |  |  |
| Oxacillin | 71 | 240 | 29.58 | \ | \ | \ | 56 | 71 | 78.87 | \ | \ | \ |
| Methicillin | \ | \ | \ | \ | \ | \ | \ | \ | \ | \ | \ | \ |
| Penicillin | \ | \ | \ | \ | \ | \ | \ | \ | \ | \ | \ | \ |
| Ampicillin | 27 | 240 | 11.25 | 6 | 139 | 4.32 | 5 | 71 | 7.04 | 9 | 20 | 45.00 |
| Vancomycin | 240 | 240 | 100.00 | 129 | 139 | 92.81 | 71 | 71 | 100.00 | 20 | 20 | 100.00 |
| Teicoplanin | \ | \ | \ | \ | \ | \ | \ | \ | \ | \ | \ | \ |
| Linezolid | \ | \ | \ | \ | \ | \ | \ | \ | \ | \ | \ | \ |
| Ciprofloxacin | 96 | 250 | 38.40 | 64 | 143 | 44.76 | 2 | 2 | 100.00 | 18 | 30 | 60.00 |
| Levofloxacin | 157 | 251 | 62.55 | 67 | 143 | 46.85 | 41 | 73 | 56.16 | 22 | 30 | 73.33 |
| Trimethoprim-sulfamethoxazole | 167 | 259 | 64.48 | \ | \ | \ | 21 | 71 | 29.58 | 12 | 20 | 60.00 |
| Rifampicin | 167 | 240 | 69.58 | 69 | 139 | 49.64 | 47 | 71 | 66.20 | 15 | 20 | 75.00 |
| Fusidic acid | \ | \ | \ | \ | \ | \ | \ | \ | \ | \ | \ | \ |
| Ampicillin/sulbactam | 6 | 11 | 54.55 | \ | \ | \ | \ | \ | \ | 4 | 10 | 40.00 |
| Tetracycline | \ | \ | \ | \ | \ | \ | \ | \ | \ | \ | \ | \ |
| Erythromycin | 130 | 270 | 48.15 | 61 | 139 | 43.88 | 31 | 73 | 42.47 | 17 | 30 | 56.67 |
| Clindamycin | 128 | 251 | 51.00 | 2 | 143 | 1.40 | 32 | 71 | 45.07 | 10 | 30 | 33.33 |
|  | | | | | | | | | | | | |
| **Europe** |  |  |  |  |  |  |  |  |  |  |  |  |
| Oxacillin | 267 | 343 | 77.84 | \ | \ | \ | \ | \ | \ | \ | \ | \ |
| Methicillin | \ | \ | \ | \ | \ | \ | \ | \ | \ | \ | \ | \ |
| Penicillin | \ | \ | \ | \ | \ | \ | 0 | 10 | 0 | 0 | 8 | 0 |
| Ampicillin | \ | \ | \ | \ | \ | \ | \ | \ | \ | \ | \ | \ |
| Vancomycin | 393 | 393 | 100.00 | 19 | 19 | 100.00 | \ | \ | \ | 16 | 16 | 100.00 |
| Teicoplanin | 372 | 372 | 100.00 | 19 | 19 | 100.00 | 6 | 10 | 60.00 | 8 | 8 | 100.00 |
| Linezolid | 26 | 26 | 100.00 | 19 | 19 | 100.00 | 10 | 10 | 100.00 | 8 | 8 | 100.00 |
| Ciprofloxacin | 257 | 367 | 70.03 | 15 | 19 | 78.95 | 6 | 10 | 60.00 | 8 | 10 | 80.00 |
| Levofloxacin | 291 | 353 | 82.44 | 11 | 19 | 57.89 | 6 | 10 | 60.00 | 16 | 16 | 100.00 |
| Trimethoprim-sulfamethoxazole | 323 | 346 | 32.26 | \ | \ | \ | \ | \ | \ | 7 | 8 | 87.50 |
| Rifampicin | 322 | 372 | 93.35 | \ | \ | \ | 9 | 10 | 90.00 | \ | \ | \ |
| Fusidic acid | \ | \ | \ | \ | \ | \ | \ | \ | \ | \ | \ | \ |
| Ampicillin/sulbactam | 0 | 21 | 0 | 11 | 11 | 100.00 | \ | \ | \ | 10 | 10 | 100.00 |
| Tetracycline | 20 | 26 | 76.92 | \ | \ | \ | 5 | 10 | 50.00 | 7 | 8 | 87.50 |
| Erythromycin | 19 | 26 | 73.08 | \ | \ | \ | 6 | 10 | 60.00 | 10 | 16 | 62.50 |
| Clindamycin | 19 | 26 | 73.08 | 0 | 8 | 0 | 5 | 10 | 50.00 | 8 | 16 | 50.00 |
|  | | | | | | | | | | | | |
| **Americas** |  |  |  |  |  |  |  |  |  |  |  |  |
| Oxacillin | 11 | 27 | 40.74 | \ | \ | \ | \ | \ | \ | 0 | 6 | 0 |
| Methicillin | \ | \ | \ | \ | \ | \ | \ | \ | \ | \ | \ | \ |
| Penicillin | 8 | 27 | 29.63 | \ | \ | \ | \ | \ | \ | 0 | 6 | 0 |
| Ampicillin | 10 | 66 | 15.15 | 2 | 5 | 40.00 | \ | \ | \ | \ | \ | \ |
| Vancomycin | 20 | 27 | 74.07 | \ | \ | \ | \ | \ | \ | 1 | 6 | 16.67 |
| Teicoplanin | \ | \ | \ | \ | \ | \ | \ | \ | \ | \ | \ | \ |
| Linezolid | \ | \ | \ | \ | \ | \ | \ | \ | \ | \ | \ | \ |
| Ciprofloxacin | 32 | 66 | 48.48 | 2 | 5 | 40.00 | \ | \ | \ | \ | \ | \ |
| Levofloxacin | \ | \ | \ | \ | \ | \ | \ | \ | \ | \ | \ | \ |
| Trimethoprim-sulfamethoxazole | 30 | 93 | 32.26 | 2 | 5 | 40.00 | \ | \ | \ | 0 | 6 | 0 |
| Rifampicin | \ | \ | \ | \ | \ | \ | \ | \ | \ | \ | \ | \ |
| Fusidic acid | \ | \ | \ | \ | \ | \ | \ | \ | \ | \ | \ | \ |
| Ampicillin/sulbactam | \ | \ | \ | \ | \ | \ | \ | \ | \ | \ | \ | \ |
| Tetracycline | 38 | 66 | 57.58 | 1 | 5 | 20.00 | \ | \ | \ | \ | \ | \ |
| Erythromycin | 9 | 27 | 33.33 | \ | \ | \ | \ | \ | \ | 0 | 6 | 0 |
| Clindamycin | \ | \ | \ | \ | \ | \ | \ | \ | \ | \ | \ | \ |

N: number of sensitive isolates, T: total number of isolates, %: percentage of sensitive isolates.

Table S11. Percentage of in vitro susceptibility to selected antibiotic agents of Gram-negative aerobic isolates from diabetic foot infections in the world.

| Antibiotic | *Escherichia coli* | | | *Klebsiella* spp. | | | *Proteus* spp. | | | *Enterobacter* spp. | | | *P. aeruginosa* | | | *Acinetobacter* spp. | | |
| --- | --- | --- | --- | --- | --- | --- | --- | --- | --- | --- | --- | --- | --- | --- | --- | --- | --- | --- |
|  | N | T | % | N | T | % | n | T | % | N | T | % | N | T | % | N | T | % |
| **Asia inc. China** |  |  |  |  |  |  |  |  |  |  |  |  |  |  |  |  |  |  |
| Amikacin | 1293 | 1760 | 73.47 | 686 | 843 | 81.38 | 982 | 1146 | 85.69 | 395 | 485 | 81.44 | 1558 | 2056 | 75.79 | 136 | 254 | 53.54 |
| Aztreonam | 540 | 1035 | 52.17 | 286 | 431 | 66.36 | 456 | 577 | 79.03 | 231 | 344 | 67.15 | 706 | 1176 | 60.03 | 44 | 94 | 46.81 |
| Cefepime | 583 | 1142 | 51.05 | 332 | 467 | 71.09 | 477 | 648 | 73.61 | 291 | 379 | 76.78 | 928 | 1351 | 68.69 | 54 | 114 | 47.37 |
| Cefoperazone-sulbactam | 585 | 784 | 74.62 | 273 | 354 | 77.12 | 251 | 314 | 79.94 | 122 | 175 | 69.71 | 697 | 1192 | 58.47 | 83 | 135 | 61.48 |
| Ceftazidime | 834 | 1615 | 51.64 | 496 | 722 | 68.70 | 737 | 932 | 79.08 | 265 | 456 | 58.11 | 1469 | 2174 | 67.57 | 88 | 192 | 45.83 |
| Cefotaxime | 427 | 1596 | 26.75 | 307 | 491 | 62.53 | 495 | 694 | 71.33 | 89 | 179 | 49.72 | 375 | 852 | 44.01 | 51 | 124 | 41.13 |
| Ciprofloxacin | 578 | 1622 | 35.64 | 571 | 878 | 65.03 | 668 | 1074 | 62.20 | 260 | 390 | 66.67 | 1426 | 2083 | 68.46 | 105 | 208 | 50.48 |
| Gentamicin | 832 | 1735 | 47.95 | 600 | 905 | 66.30 | 655 | 1066 | 61.44 | 290 | 462 | 62.77 | 1295 | 2061 | 62.83 | 70 | 195 | 35.90 |
| Imipenem | 1558 | 1683 | 92.57 | 832 | 892 | 93.27 | 951 | 1052 | 90.40 | 448 | 490 | 91.43 | 1758 | 2103 | 83.59 | 152 | 218 | 69.72 |
| Levofloxacin | 531 | 1326 | 40.05 | 363 | 505 | 71.88 | 469 | 670 | 70.00 | 209 | 356 | 58.71 | 766 | 1232 | 62.18 | 108 | 199 | 54.27 |
| Trimethoprim-sulfamethoxazole | 258 | 796 | 32.41 | 168 | 369 | 45.53 | 283 | 671 | 42.18 | 141 | 285 | 49.47 | 102 | 558 | 18.20 | 63 | 188 | 33.51 |
| Piperacillin/tazobactam | 1006 | 1316 | 76.44 | 425 | 536 | 79.29 | 649 | 823 | 78.86 | 328 | 425 | 77.17 | 1273 | 1689 | 75.37 | 104 | 233 | 44.64 |
| Ampicillin | 198 | 963 | 20.56 | 147 | 495 | 29.70 | 333 | 767 | 43.42 | 54 | 352 | 15.34 | 211 | 476 | 44.33 | 9 | 47 | 19.15 |
| Ampicillin-sulbactam | 236 | 509 | 46.37 | 261 | 381 | 68.50 | 290 | 482 | 60.17 | 77 | 142 | 54.23 | 90 | 204 | 44.12 | 71 | 125 | 56.80 |
|  | | | | | | | | | | | | | | | | | | |
| **Africa** |  |  |  |  |  |  |  |  |  |  |  |  |  |  |  |  |  |  |
| Amikacin | 39 | 86 | 45.35 | 54 | 81 | 66.67 | 25 | 72 | 34.72 | 18 | 25 | 72.00 | 69 | 102 | 67.65 | 19 | 25 | 76.00 |
| Aztreonam | 42 | 86 | 48.84 | 67 | 81 | 82.72 | 61 | 72 | 84.72 | 21 | 25 | 84.00 | \ | \ | \ | 19 | 25 | 76.00 |
| Cefepime | \ | \ | \ | \ | \ | \ | \ | \ | \ | \ | \ | \ | \ | \ | \ | \ | \ | \ |
| Cefoperazone-sulbactam | \ | \ | \ | \ | \ | \ | \ | \ | \ | \ | \ | \ | \ | \ | \ | \ | \ | \ |
| Ceftazidime | 53 | 86 | 61.63 | 34 | 81 | 41.98 | 26 | 72 | 36.11 | 11 | 25 | 44.00 | 63 | 102 | 61.76 | 13 | 25 | 52.00 |
| Cefotaxime | 39 | 86 | 45.35 | 54 | 81 | 66.67 | 25 | 72 | 34.72 | 13 | 25 | 52.00 | 62 | 102 | 60.78 | 19 | 25 | 76.00 |
| Ciprofloxacin | 47 | 98 | 47.96 | 53 | 86 | 61.63 | 67 | 93 | 72.04 | 20 | 25 | 80.00 | 80 | 106 | 75.47 | 21 | 25 | 84.00 |
| Gentamicin | 47 | 98 | 47.96 | 34 | 86 | 39.53 | 67 | 93 | 72.04 | 16 | 25 | 64.00 | 64 | 106 | 60.38 | 2 | 19 | 10.53 |
| Imipenem | 86 | 86 | 100.00 | 80 | 81 | 98.77 | 70 | 72 | 97.22 | 24 | 25 | 96.00 | 99 | 102 | 97.06 | 14 | 37 | 37.84 |
| Levofloxacin | 34 | 86 | 39.53 | 67 | 81 | 82.72 | 52 | 72 | 72.22 | 19 | 25 | 76.00 | 89 | 102 | 87.26 | \ | \ | \ |
| Trimethoprim-sulfamethoxazole | 33 | 98 | 33.67 | 36 | 86 | 41.86 | 31 | 84 | 36.90 | 6 | 25 | 24.00 | 0 | 4 | 0 | 13 | 25 | 52.00 |
| Piperacillin/tazobactam | 33 | 86 | 38.37 | 53 | 81 | 65.43 | 37 | 72 | 51.39 | 20 | 25 | 80.00 | 73 | 102 | 71.57 | 19 | 25 | 76.00 |
| Ampicillin | 19 | 86 | 22.09 | 5 | 81 | 6.17 | 42 | 81 | 51.85 | \ | \ | \ | \ | \ | \ | 9 | 25 | \ |
| Ampicillin-sulbactam | \ | \ | \ | \ | \ | \ | \ | \ | \ | \ | \ | \ | \ | \ | \ | \ | \ | \ |
|  | | | | | | | | | | | | | | | | | | |
| **Europe** |  |  |  |  |  |  |  |  |  |  |  |  |  |  |  |  |  |  |
| Amikacin | 7 | 12 | 58.33 | \ | \ | \ | 2 | 4 | 50.00 | 267 | 272 | 98.16 | 154 | 184 | 83.70 | \ | \ | \ |
| Aztreonam | \ | \ | / | \ | \ | \ | \ | \ | \ | 229 | 272 | 84.19 | 90 | 126 | 71.43 | \ | \ | \ |
| Cefepime | \ | \ | / | \ | \ | \ | \ | \ | \ | 229 | 272 | 84.19 | 100 | 126 | 79.37 | \ | \ | \ |
| Cefoperazone-sulbactam | 5 | 12 | 41.67 | \ | \ | \ | 3 | 4 | 75.00 | \ | \ | \ | 23 | 58 | 39.66 | \ | \ | \ |
| Ceftazidime | 15 | 20 | 75.00 | 11 | 12 | 91.67 | 6 | 11 | 54.55 | 240 | 281 | 85.41 | 151 | 195 | 77.44 | \ | \ | \ |
| Cefotaxime | 8 | 12 | 66.67 | \ | \ | \ | 4 | 4 | 100 | 231 | 272 | 84.93 | 28 | 58 | 48.28 | \ | \ | \ |
| Ciprofloxacin | 15 | 20 | 75.00 | 11 | 12 | 91.67 | 8 | 11 | 72.73 | 229 | 281 | 81.49 | 115 | 195 | 58.97 | \ | \ | \ |
| Gentamicin | 6 | 12 | 50.00 | \ | \ | \ | 3 | 4 | 75.00 | 245 | 272 | 90.07 | 119 | 184 | 64.67 | \ | \ | \ |
| Imipenem | 8 | 8 | 100.00 | 12 | 12 | 100 | 5 | 7 | 71.43 | 276 | 281 | 98.22 | 103 | 137 | 75.18 | \ | \ | \ |
| Levofloxacin | 11 | 20 | 55.00 | 11 | 12 | 91.67 | 9 | 11 | 81.82 | 234 | 281 | 83.27 | 117 | 125 | 93.6 | \ | \ | \ |
| Trimethoprim-sulfamethoxazole | 6 | 8 | 75.00 | 10 | 12 | 83.33 | 2 | 7 | 28.57 | 193 | 281 | 68.68 | 1 | 11 | 9.09 | \ | \ | \ |
| Piperacillin/tazobactam | 8 | 8 | 100.00 | 12 | 12 | 100 | 6 | 7 | 85.71 | 265 | 281 | 94.31 | 112 | 137 | 81.75 | \ | \ | \ |
| Ampicillin | 0 | 12 | 0 | \ | \ | \ | 0 | 4 | 0 | 46 | 272 | 16.91 | 0 | 58 | 0 | \ | \ | \ |
| Ampicillin-sulbactam | \ | \ | \ | \ | \ | \ | \ | \ | \ | \ | \ | \ | \ | \ | \ | \ | \ | \ |
|  | | | | | | | | | | | | | | | | | | |
| **Americas** |  |  |  |  |  |  |  |  |  |  |  |  |  |  |  |  |  |  |
| Amikacin | \ | \ | \ | \ | \ | \ | \ | \ | \ | \ | \ | \ | \ | \ | \ | \ | \ | \ |
| Aztreonam | 2 | 4 | 50.00 | \ | \ | \ | 4 | 10 | 40.00 | 2 | 9 | 22.22 | 1 | 4 | 25.00 | \ | \ | \ |
| Cefepime | \ | \ | \ | \ | \ | \ | \ | \ | \ | \ | \ | \ | \ | \ | \ | \ | \ | \ |
| Cefoperazone-sulbactam | \ | \ | \ | \ | \ | \ | \ | \ | \ | \ | \ | \ | \ | \ | \ | \ | \ | \ |
| Ceftazidime | 0 | 6 | 0 | 19 | 36 | 52.78 | 18 | 23 | 78.26 | 16 | 20 | 80.00 | 10 | 25 | 40.00 | 20 | 26 | 76.92 |
| Cefotaxime | 2 | 4 | 50.00 | \ | \ | \ | 4 | 10 | 40.00 | 1 | 9 | 11.11 | 1 | 4 | 25.00 | \ | \ | \ |
| Ciprofloxacin | 6 | 6 | 100.00 | 32 | 36 | 88.89 | 14 | 23 | 60.87 | 11 | 20 | 55.00 | 11 | 25 | 44.00 | 18 | 26 | 69.23 |
| Gentamicin | 9 | 10 | 90.00 | 25 | 36 | 69.44 | 23 | 33 | 69.70 | 14 | 29 | 48.28 | 23 | 29 | 79.31 | 15 | 26 | 57.69 |
| Imipenem | 4 | 10 | 40.00 | 32 | 36 | 88.89 | 26 | 33 | 78.79 | 18 | 27 | 66.67 | 23 | 29 | 79.31 | 24 | 26 | 92.31 |
| Levofloxacin | \ | \ | \ | \ | \ | \ | \ | \ | \ | \ | \ | \ | \ | \ | \ | \ | \ | \ |
| Trimethoprim-sulfamethoxazole | 0 | 6 | 0 | 28 | 36 | 77.78 | 12 | 33 | 36.36 | 11 | 20 | 55.00 | 15 | 25 | 60.00 | 20 | 26 | 76.92 |
| Piperacillin/tazobactam | 5 | 6 | 83.33 | 17 | 36 | 47.22 | 18 | 23 | 78.26 | 15 | 20 | 75.00 | 16 | 25 | 64.00 | 21 | 26 | 80.77 |
| Ampicillin | 1 | 10 | 10.00 | 0 | 36 | 0 | 9 | 33 | 27.27 | 0 | 29 | 0 | 0 | 29 | 0 | 0 | 26 | 0 |
| Ampicillin-sulbactam | \ | \ | \ | \ | \ | \ | \ | \ | \ | \ | \ | \ | \ | \ | \ | \ | \ | \ |

N: number of sensitive isolates, T: total number of isolates, %: percentage of sensitive isolates.
