# Supplementary material for: Microbiological profile of diabetic foot infections in China and worldwide: a 20-year systematic review
Source: Front Endocrinol (Lausanne). 2024 Jun 28;15:1368046. doi: 10.3389/fendo.2024.1368046 (PMC11247326; doi:10.3389/fendo.2024.1368046)
Supplement: Supplementary file 3 [file DataSheet_1.docx]

**Supplementary File**

**1. Distribution of Microorganisms in Different Asian Regions**

Based on the theory that antibiotic selection is informed by the type of microbiological profile in Asia, this study examined the composition and susceptibility of bacterial isolate in diabetic foot infection (DFI) samples from Asian centers. Using 315 Asian studies, we evaluated 54,161 tissue and bone samples from 48,241 individuals.

Overall, the incidence of GPB and Fungi in other Asian countries compared with that in China (GPB: 44.82% and 46.58%, respectively; Fungi: 3.78% and 4.68%, respectively) was lower. The incidence of GNB and obligate anaerobes in other Asian countries compared with that in China (GNB: 49.36 and 47.18%, respectively; 2.04% and 1.56%, respectively) tended to be higher (Figure 6). Notably, MRSA isolation rates were significantly lower in China than in other Asian countries (8.24 and 12.82%, respectively).

Asia is divided based on physical geography into Eastern, Southeast, South Central, and Western Asia regions. The incidence of GPB in Eastern Asia (including China) was the highest (46.84%), whereas GPB incidence in Southeast Asia was the lowest (30.61%); however, MRSA incidence in Southeast Asia tended to be the highest (43.29%). GNB incidence (61.32%) in Southeast Asia tended to be the highest. The incidence of *E. coli* (15.74%) in South Central Asia and *Klebsiella spp.* (11.11%), *Proteus spp.* (10.85%), *Enterobacter spp.* (6.87%), *Pseudomonas aeruginosa* (16.46%), and *Acinetobacter spp.* (2.62%) in Southeast Asia tended to be the highest. Moreover, obligate anaerobe incidence in Southeast Asia trended to be the highest (7.60%), and fungal species incidence in Eastern Asia (including China) tended to be the highest (4.58%) (Figure 6 and Table S6).

**2.** **Distribution and Antibiotic Susceptibility of Microorganisms by Regions**

Based on DFI samples from different centers worldwide, we analyzed the global bacterial isolate profile from 2000–2020. Asian microbiological profile differs from that of other continents and should inform empirical antimicrobial selection. Between 2000 and 2020, 359 studies reported 56,592 cases (38,744 from China) and 67,151 bacterial isolates (41,427 from China).

The isolation rates of GPB in DFI were greatest in America (62.74%) and lowest in Asia, including China (44.82%). Africa had the most isolated MRSA (26.90%). GNB (49.36%) were mostly common in Asia. In Asia, *E. coli* (10.77%), *Enterobacter spp.* (3.95%), *P. aeruginosa* (11.08%), *Acinetobacter Spp.* (2.52%), and *Klebsiella* *Spp.* (7.54%) were the most common. In Africa, *Klebsiella Spp.* (11.11%) and *Proteus Spp.* (9.10%) were the most common. The incidence of obligate anaerobe and fungi was highest in Africa (11.42%) and Asia (3.78%), respectively (Figure 7 and Table S7).

**3. Worldwide Antibiotic Susceptibility Patterns of Isolates Obtained from DFIs**

Based on the assumption that the global microbiological profile affects antimicrobial selection, this research examined antibiotic susceptibility trends in DFI samples from different centers worldwide. Table S9 presents antibiotic susceptibility data of all GOB isolates. Vancomycin was the most effective against GPB in Asia (98.05%), Africa (100%), Europe (100%), and America (74.07%). Vancomycin was the most effective against *Enterococcus spp.* in Asia (91.96%), Africa (92.81%), and Europe (100%). Vancomycin was the most effective against CoNS, with 96.19% and 100% sensitive strains in Asia and Africa, respectively. Vancomycin was the most effective against *Streptococcus spp*., with 94.83%, 100%, and 16.67% susceptible strains in Asia, Europe, and America, respectively.

Table S10 presents GNB antimicrobial sensitivity values. No antibiotic was 100% effective against GNBs; however, imipenem was the most effective against *E. coli*, with 92.57%, 100%, and 40% susceptible strains in Asia, Africa, Europe, and America, respectively. Imipenem was the most effective against *Klebsiella spp*., with 93.27%, 98.77%, 100%, and 88.77% susceptible strains in Asia, Africa, Europe, and America, respectively. Imipenem was the most effective against *Proteus spp.*, with 90.40%, 97.22%, 71.43%, and 78.79% susceptible strains in Asia, Africa, Europe, and America, respectively. Imipenem was the most effective against *Enterobacter spp*., with 91.43%, 96.00%, 98.22%, and 66.67% of DFI-susceptible strains in Asia, Africa, Europe, and America, respectively. Impenem was the most effective against *P. aeruginosa*, with 83.59%, 97.06%, 75.18%, and 79.31% strains susceptible strains in Asia, Africa, Europe, and America, respectively. Imipenem was effective against *Acinetobacter spp.,* with 69.72%, 37.84%, and 92.315 strains in Asia, Africa, and America, respectively.
